# Mod-SE(2): a geometric deep learning framework for brain tumor classification and segmentation in MRI images

**DOI:** 10.1186/s12929-025-01213-y

**Published:** 2026-01-12

**Authors:** Clara Lavita Angelina, Fu-Ren Xiao, Sunil Vyas, Pan-Chyr Yang, Hsuan-Ting Chang, Yuan Luo

**Affiliations:** 1 Graduate School of Engineering Science and Technology, National Yunlin University of Science and Technology, Yunlin, 64002 Taiwan; 2 Department of Electrical Engineering, National Yunlin University of Science and Technology, Yunlin, 64002 Taiwan; 3 Institute of Medical Device and Imaging, National Taiwan University, Taipei, 10051 Taiwan; 4 Department of Internal Medicine, National Taiwan University Hospital, Taipei, 100225 Taiwan; 5 College of Medicine, National Taiwan University Hospital, National Taiwan University, Taipei, 10051 Taiwan; 6 Graduate School of Intelligent Data Science, National Yunlin University of Science and Technology, Yunlin, 64002 Taiwan; 7 YongLin Institute of Health, National Taiwan University, Taipei, 10087 Taiwan; 8 Department of Biomedical Engineering, National Taiwan University, Taipei, 10617 Taiwan; 9 Taiwan International Bio Research Center, National Taiwan University, Taipei, 106319 Taiwan; 10 Program for Precision Health and Intelligent Medicine, National Taiwan University, Taipei, 106319 Taiwan

**Keywords:** Geometric deep learning, Mod-SE(2), Brain tumor classification, MRI, Medical imaging, Roto-translation invariance

## Abstract

**Background:**

Accurate classification and segmentation of brain tumors in MRI scans are essential for diagnosis and treatment planning. However, the heterogeneous morphology of brain tumors, including irregular shapes, sizes, and spatial variability, makes this task highly challenging. Traditional convolutional neural networks (CNNs) lack rotational and translational invariance, which limits their ability to generalize across different orientations.

**Methods:**

This study introduces a geometric deep learning framework called Modified Special Euclidean (Mod-SE(2)), which integrates geometric priors to enhance spatial consistency and reduce reliance on data augmentation. By incorporating symmetry-preserving group convolutions and spatial priors, Mod-SE(2) improves the robustness in tumor classification (namely Mod-Cls-SE(2)) and segmentation (mentioned as Mod-Seg-SE(2)). Unlike conventional CNNs, geometric deep learning encodes roto-translation symmetry directly into the architecture. This addresses the spatial variability and orientation sensitivity that are common in MRI-based diagnostics. Mod-SE(2) was evaluated on three MRI datasets and two other medical image datasets for classification and segmentation tasks. It incorporates lifting layers, group convolutions, and feature recalibration. It was benchmarked against U-Net, NN U-Net, VGG16, VGG19, and ResNet architectures.

**Results:**

Mod-Cls-SE(2) achieved an average classification accuracy of 0.914, outperforming ResNet101 with 0.682, VGG16 with 0.705, and their variants. In the binary classification of five tumor types (AVM, Meningioma, Pituitary, Metastases, and Schwannoma) from the private dataset, the model achieved an accuracy of 0.935 and a precision of 0.960 for pituitary tumors and a precision of 0.96. For segmentation tasks, Mod-Seg-SE(2) achieved a dice coefficient of 0.9503 and an IoU of 0.9616 on the BraTS2020 dataset. This result exceeds those of U-Net and NN U-Net with dice scores of 0.797 and 0.815, respectively. The model also reduced inference time and demonstrated strong computational performance.

**Conclusions:**

Mod-SE(2) uses geometric priors to improve the spatial consistency, efficiency, and interpretability in brain tumor analysis. Its symmetry-aware design enables better generalization across tumor shapes and outperforms traditional methods across all key metrics. The Mod-SE(2) CNN ensures accurate boundary delineation, supporting neurosurgical planning, intraoperative navigation, and downstream applications such as Monte Carlo-based radiotherapy simulations and PET-MRI co-registration. Future work will extend the model to 3D volumes and validate its clinical readiness.

**Supplementary Information:**

The online version contains supplementary material available at 10.1186/s12929-025-01213-y.

## Introduction

Brain tumors pose a significant challenge in clinical practice due to their diverse appearances, growth patterns, and locations within the brain [1]. Among the various intracranial growths encountered, arteriovenous malformations (AVMs), pituitary tumors, meningiomas, schwannomas, and metastatic lesions account for a large percentage of diagnosed cases. Although Magnetic Resonance Imaging (MRI) is the established standard for evaluating brain tumors, providing high-resolution details of soft tissues and anatomy [2], distinguishing between different tumor types can be difficult to distinguish. The precise classification and segmentation of these tumors remains challenging due to overlapping features seen on imaging and the variability within individual tumors [3, 4]. The necessity of precise diagnostic tools that can accurately differentiate between these tumor types underscores the importance of innovations in medical imaging technology [5, 6]. While histopathology and molecular analysis remain essential for definitive diagnosis, prognostication, and treatment planning in neuro-oncology, these methods are time-consuming, require invasive procedures, and are not always practical in urgent clinical situations. These limitations have led to a growing interest in automated, high-precision diagnostic tools that can deliver reliable results with minimal human intervention. In this work, we aim to enhance non-invasive imaging analysis prior to histopathology.

Each type of brain tumor presents unique challenges for both imaging and biological interpretation. AVMs, being congenital vascular abnormalities, have complex, tangled vessel structures that make them difficult to outline precisely. Pituitary tumors, though often small, exhibit a wide range of functional profiles that impact hormone levels and thus complicate diagnosis. Meningiomas and schwannomas can appear similar on MRI scans despite originating from different cell types. The diverse imaging characteristics of metastases, which depend on their primary source, add another layer of complexity. Accurately classifying and delineating these various tumor types is crucial for effective treatment planning, guiding surgical procedures, and predicting patient outcomes.

Convolutional neural networks (CNNs) have shown strong performance in both classification and segmentation tasks, including lung cancer classification [7] and brain tumor analysis [8–10]. Notably, models like Res-BRNet and EfficientNet-B0 have achieved impressive results when trained on large-scale datasets [11]. Similarly, architectures such as U-Net [12] and NN U-Net [13] have become the standard for tumor segmentation tasks. DeepLabV3+ and Efficient-Enhanced U-Net also achieved impressive results when trained on BraTS dataset [14, 15]. However, CNNs inherently struggle with spatial variations, especially rotations and translations. This requires extensive data augmentation, which can lead to inconsistencies in how features are extracted from MRIs acquired in different orientations. Consequently, this increases computational demands and reduces the interpretability of the models. Although transfer learning has improved performance in some instances [16], pre-trained models still lack an inherent understanding of geometric invariance.

To overcome these limitations, geometric deep learning (GDL) has emerged as a promising alternative. Models such as Group Equivariant CNNs (G-CNNs) [17] and Harmonic Networks [18] are designed to respect rotational symmetry, making them more robust to changes in orientation. However, many of these models primarily focus on rotational equivariance and often overlook translational invariance, which is vital for accurately locating tumors. Previous research has typically concentrated on either classification or segmentation, rarely addressing both tasks within a unified model or encompassing the full spectrum of brain tumor diversity.

GDL provides a biologically inspired approach to incorporating spatial knowledge and structural symmetry into neural networks [19–21]. It has gained increasing traction across medical imaging domains beyond brain tumor classification. For instance, Bronstein et al. demonstrated the GDL in analyzing cortical surfaces and mapping functional regions using non-Euclidean mesh representations [22]. In diffusion MRI, Ewert et al. introduced a GDL method for continuous signal reconstruction [23]. A deeper representation of GDL is also employed. Zhang et al. applied local-to-global graph neural networks to classify Autism Spectrum Disorder (ASD) and Alzheimer’s disease (AD) by analyzing disease-related local brain regions and biomarkers [24]. Additionally, Kerkela et al. modeled spherical neural networks to improve the estimation of microstructural data from brain MRI data [25]. This research improves prediction accuracy compared to models that apply less or no rotational variance. This emphasizes the effectiveness of a model in medical images for models that use rotational features, such as GDL methods. Zhu et al. proposed a unified GDL model to predict task-based fMRI activity from anatomical features in sMRI, highlighting cross-modal learning [26]. In CT imaging, Koetzier et al. presented a graph-based deep reconstruction method to recover high-fidelity CT volumes from undersampled data [27]. Winkels and Cohen proved CNN model with roto-translation group convolutions performs similarly to a regular CNN with ten times less data [28]. This research trained a roto-translation group 3D CNN on a pulmonary nodule detection dataset and compared it with a regular CNN model. The regular CNN model received data augmentation, yet still needed ten times more data to perform at an acceptable level. In digital pathology, Li et al. use GDL to conduct spatial analysis using histopathology images for single-cell analysis [29]. Biologically, analyzing histopathology images at the single-cell level offers a significant advantage over traditional patch-based methods by providing detailed cellular information. This research focuses on exploring more into the microenvironment of a tumor. In their experiments, their proposed model has consistently eclipsed existing patch-based and cell graph-based methods across two independent datasets.

This study aims to address two fundamental tasks in neuro-oncological imaging, which are classifying various tumor types and performing semantic segmentation. For classification, we use a private MRI dataset containing five distinct tumor categories: AVM, meningioma, pituitary adenoma, metastatic tumors, and schwannoma. Arteriovenous malformations are vascular malformations with an incidence of approximately 1 per 100,000 per year and carry a risk of serious hemorrhage, where their detection via MRI is critical for preventing stroke and guiding surgical planning [30, 31]. Meningiomas account for roughly 30-37 % of all primary brain tumors, are frequently benign, and often produce symptoms through mass effect, with MRI guiding both diagnosis and surgical strategy [32]. Pituitary adenomas represent 10–25% of intracranial neoplasms, with microadenomas being particularly common (prevalence up to ~17%), and can cause visual or hormonal symptoms that MRI is uniquely suited to identify [33]. Brain metastases, occurring in an estimated 10–40 % of cancer patients, are the most frequent intracranial tumors, significantly impacting prognosis and treatment decisions [34]. Vestibular schwannomas (also called acoustic neuromas) are benign tumors arising from Schwann cells of the vestibular portion of the eighth cranial nerve. They account for approximately 8–10% of all intracranial tumors and up to 80% of tumors in the cerebellopontine angle. Patients typically present with hearing loss, tinnitus, and balance disturbances [35]. Collectively, these tumor types underscore the need for an imaging tool capable of both classification and localization across heterogeneous tumor morphologies in real-world neuro-oncology practice. For segmentation, we perform pixel-level labeling to precisely outline tumor regions. This is essential for estimating tumor volume and tracking its boundaries. Our goal is to provide radiologists with clinically valuable insights to assist them in diagnosis and treatment planning.

To achieve these goals, we introduce Mod-SE(2), a Modified Special Euclidean CNN that combines roto-translational equivariance with a mechanism for recalibrating feature importance. This approach ensures spatial consistency, enabling the model to robustly classify and segment brain tumors while reducing the need for extensive data augmentation. By integrating GDL, our approach leverages spatial relationships and structural priors. The GDL approach can significantly improve both classification accuracy and segmentation precision [36]. Our primary contributions include: (1) developing Mod-SE(2), an extension of SE(2) CNNs that explicitly accounts for both rotational and translational transformations (2) demonstrating improved performance compared to conventional models using both publicly available and our private datasets and (3) analyzing computational efficiency and highlighting its reduced processing cost and improved scalability. From a clinical standpoint, Mod-SE(2) allows for a more interpretable and reproducible tumor analysis. Different tumor types, such as AVMs, schwannomas, and meningiomas, not only have distinct origins and exhibit characteristic spatial behaviors. By preserving these geometric structures, Mod-SE(2) makes predictions that more accurately reflect the actual anatomy. These predictions can aid in tasks such as surgical planning and monitoring tumor progression over time. Furthermore, the model’s ability to precisely map boundaries and provide high-resolution structural priors enhances interpretation and tumor localization. This enables neurosurgeons to plan resections while preserving functional brain regions. Its ability to reduce processing time while maintaining accuracy makes Mod-SE(2) a practical tool for real-time neurodiagnostic workflows. Our model is versatile and clinically relevant across the full spectrum of neuro-oncology care.

## Methodology

This section explains the building blocks of the CNN-based Modified Special Euclidean (Mod-SE(2)) architecture. It highlights the core components that define this convolutional network, which is based on a modification of the Special Euclidean group SE(2). As part of this modification, we replaced the max-pooling layers in the baseline SE(2)-CNN with average pooling, as preliminary experiments showed that max pooling suppressed fine-scale activations critical for detecting small or morphologically complex tumors. Average pooling preserved these subtle yet informative signals, resulting in more stable spatial representations and improved robustness across both classification and segmentation settings. Specifically, it introduces two specialized variants, which are Mod-Cls-SE(2) for classification tasks and Mod-Seg-SE(2) for segmentation tasks. Additionally, this section outlines the evaluation metrics used to assess the robustness of the proposed models, or how well they perform or remain stable under specific conditions, such as geometric transformations.

### Model architecture and training

#### Mathematical Formulation of Mod-SE(2)

The proposed Mod-SE(2) ensures that the corresponding transformations in feature maps are preserved throughout the network when rotational and translational transformations are applied to the input. Mathematically, this is formulated as follows:

1 $${T}_{g}(f(x))=f({T}_{g}(x))$$

where $f\left(x\right)$ represents the feature map that encodes tumor characteristics, and ${T}_{g}$ a Mod-SE(2) transformation (e.g., rotation, or translation). Unlike standard CNNs, which require data augmentation, Mod-SE(2) group convolutions inherently encode these transformations, ensuring robustness across varying orientations.

**Fig. 1:**
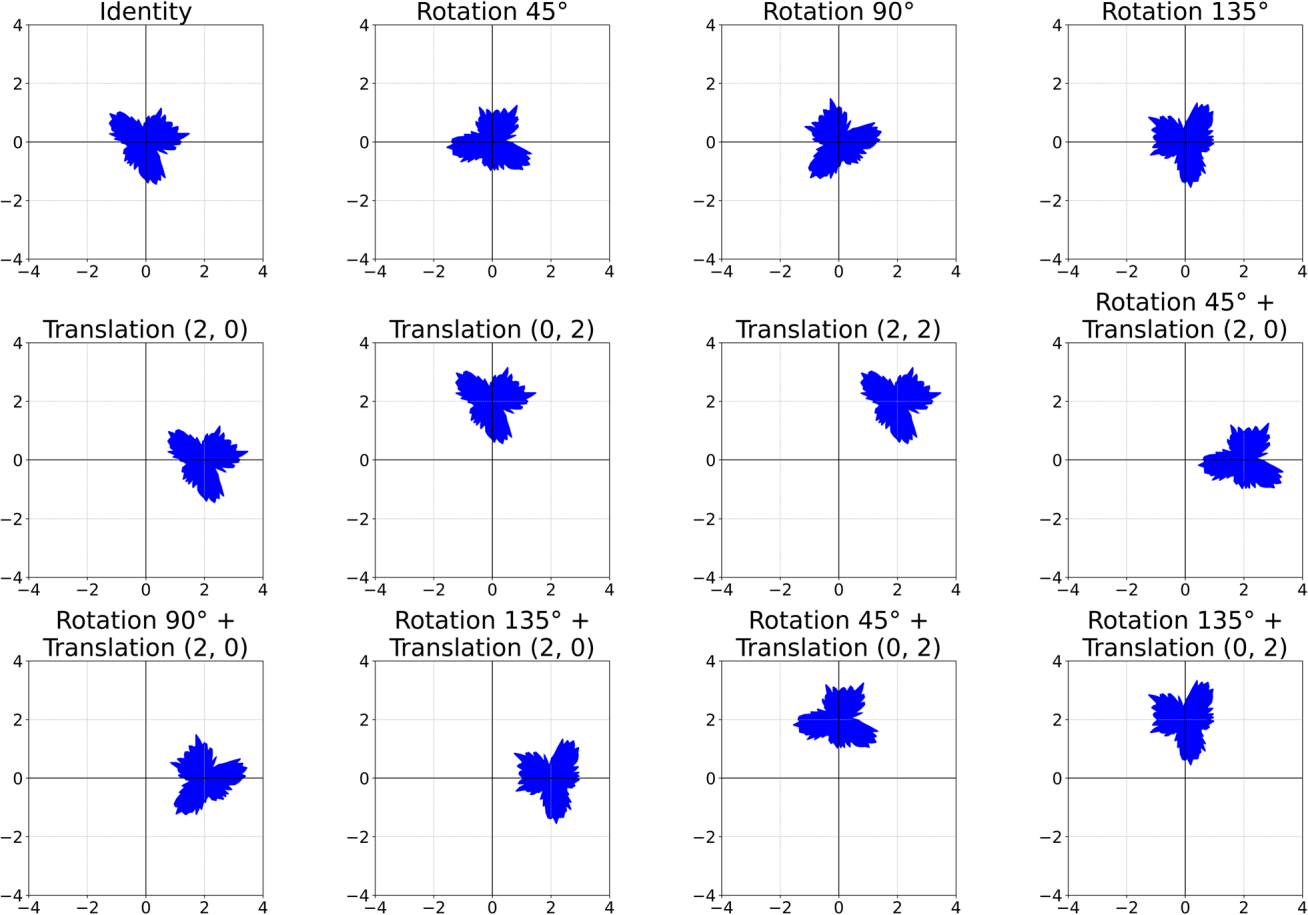
Visualization of Mod-SE(2) transformations applied to a biological object, illustrating rotations, translations, and their combinations

To maintain high-level stability, deeper layers enforce invariance,

2 $$f({T}_{g}(x))= f(x)$$

where group pooling aggregates features across transformations, thereby forming figure representations. Figure 1 shows the transformation visually. It illustrates a series of geometric transformations applied to a reference shape. These transformations include rotations, translations, and combinations of both. All this transformation applied to the original shape, which is located at the center (Identity). Mathematically, the group action of SE(2) on a point $x \in {\mathbb{R}}_{2}$ is defined as:

3 $${T}_{\left(\theta ,t\right)}\left(x\right)= {R}_{\theta }x + t$$

where ${R}_{\theta }$ is a rotation matrix, and $t$ a translation vector, reflecting the SE(2) group structure. For an element ${g=(x, R}_{\theta })$∈ Mod-SE(2), transformations act as:

4 $${T}_{g}(I) = {R}_{\theta } \cdot I + t$$

where $I$ is an original input image. With equivariance in intermediate layers:

5 $${T}_{g}(f(I)) = f({T}_{g}(I))$$

And invariance in classification:

6 $$f({T}_{g}(I)) = f(I)$$

The Mod-SE(2) group models spatial transformations in 2D, combining rotation and translation [37]. Each element g can be re ′ presented as a pair ${(x, R}_{\theta },$ where $x$ denotes a translation vector (spatial shift for aligning anatomical structures), and ${R}_{\theta }$ is a rotation matrix for angle $\theta$, enabling orientation-invariant feature learning.

7 $$g . g\prime = \left( {x, R_{\theta } } \right)\cdot \left( {x\prime , R_{{\theta^{\prime}}} } \right) = \left( {R_{\theta } x\prime + x, R_{\theta + \theta \prime } } \right)$$

This composition allows the Mod-SE(2) CNN to propagate spatial transformations coherently across network layers, ensuring consistent boundary localization and internal textures under varying orientations. This is an important step to achieve reliable segmentation.

#### Network architecture

The Mod-SE(2) model was used for classification and segmentation. It incorporates roto-translation layers and group convolutions, which improve spatial feature extraction. These enhancements make the model particularly effective in medical imaging, where anatomical structures exhibit orientation variability [17].

To clarify in subsequent sections, we refer to the classification-specific configuration of the model as Mod-Cls, and the segmentation-specific configuration as Mod-Seg. Mod-Cls is optimized to identify and categorize image-level features, while the Mod-Seg configuration is tailored for pixel-level delineation of anatomical regions.

#### Classification network

The core architecture is adapted from the original SE(2) group convolutional network proposed by [17], with custom modifications tailored to our dataset. Specifically, we replaced the original max pooling layers with average pooling. Average pooling resulted in smoother downsampling of features and improved the stability of the network when processing the high-resolution MRI scans in our dataset. Additionally, we fine-tuned the number of filters used in each layer and adjusted the dimensions of the intermediate feature representations to better align with the characteristics of our datasets, including their distribution, resolution, and inherent variability, of our datasets.

**Fig. 2:**
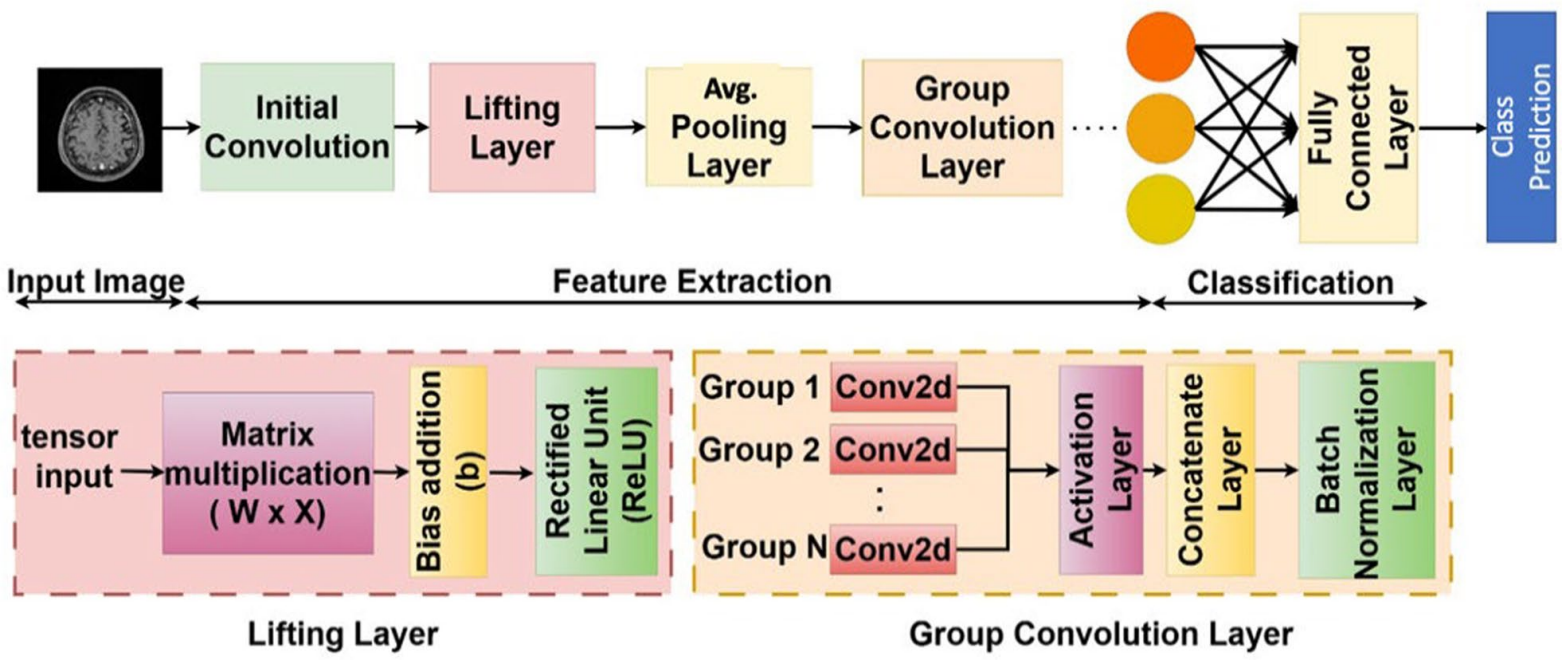
Mod-Cls-SE(2) architecture for brain tumor classification. The network includes an initial convolution layer, a lifting layer for roto-translation, group convolutions for rotation equivariance, and average pooling. It concludes with a fully connected layer to classify various tumor types

Figure 2 shows the classification pipeline of Mod-Cls-SE(2), which begins with a Conv2D layer (3 × 3 kernel, 32 filters), followed by the Lifting Layer (64 filters). The lifting layer plays a critical role by transforming the standard 2D image data, which exists on the Euclidean plane ${\mathbb{R}}^{2}$, into a richer, higher-dimensional representation on the SE(2) group. This abstract space inherently captures both translational and discrete rotational transformations. This transformation is achieved by convolving the input image with a set of filters, each rotated at different angles (for instance, 8 or 16 distinct orientations). This process generates a set of feature maps that are oriented according to the features they detect. By encoding features based on both their location and their orientation, the lifting layer preserves crucial spatial structure and directional information, setting the stage for subsequent layers to perform equivariant processing. This joint encoding of position and orientation allows the network to differentiate between patterns that might look structurally similar but appear at different angles – a particularly important capability in the context of medical image analysis.

Following the lifting layer, the architecture employs a sequence of group convolution layers. These layers perform convolutions directly within the SE(2) group domain. Unlike standard convolutional layers, which are only equivariant to translations (meaning a shift in the input leads to a corresponding shift in the output), group convolutions maintain equivariance to both rotations and translations. This means that if the input image (or the features it contains) is shifted or rotated, the resulting output feature maps will transform consistently and predictably. This property makes our network significantly more robust to the natural anatomical variability. Consequently, this inherent geometric awareness reduces the need for extensive data augmentation during training and ultimately improves the model’s generalization performance on unseen data.

To reduce the spatial dimensions and extract dominant features, we incorporate an average pooling layer after the lifting layer. Unlike max pooling, which captures only the most prominent activation in each region, average pooling summarizes features by computing their mean and preserving broader contextual information. After each group convolution, we apply batch normalization to stabilize training and a Squeeze-and-Excitation (SE) block to enable the network to learn channel-wise attention, effectively weighing the importance of different feature channels. The resulting feature maps are then flattened and fed into a series of fully connected layers, culminating in a Softmax classifier that outputs the probability of each tumor class. Mod-Cls-SE(2) integrates roto-translation transformations, enabling robust handling of spatial variations in medical images (see Section 1: Mod-Cls-SE(2) Architecture in Supplementary File 1).

#### Segmentation network

**Fig. 3:**
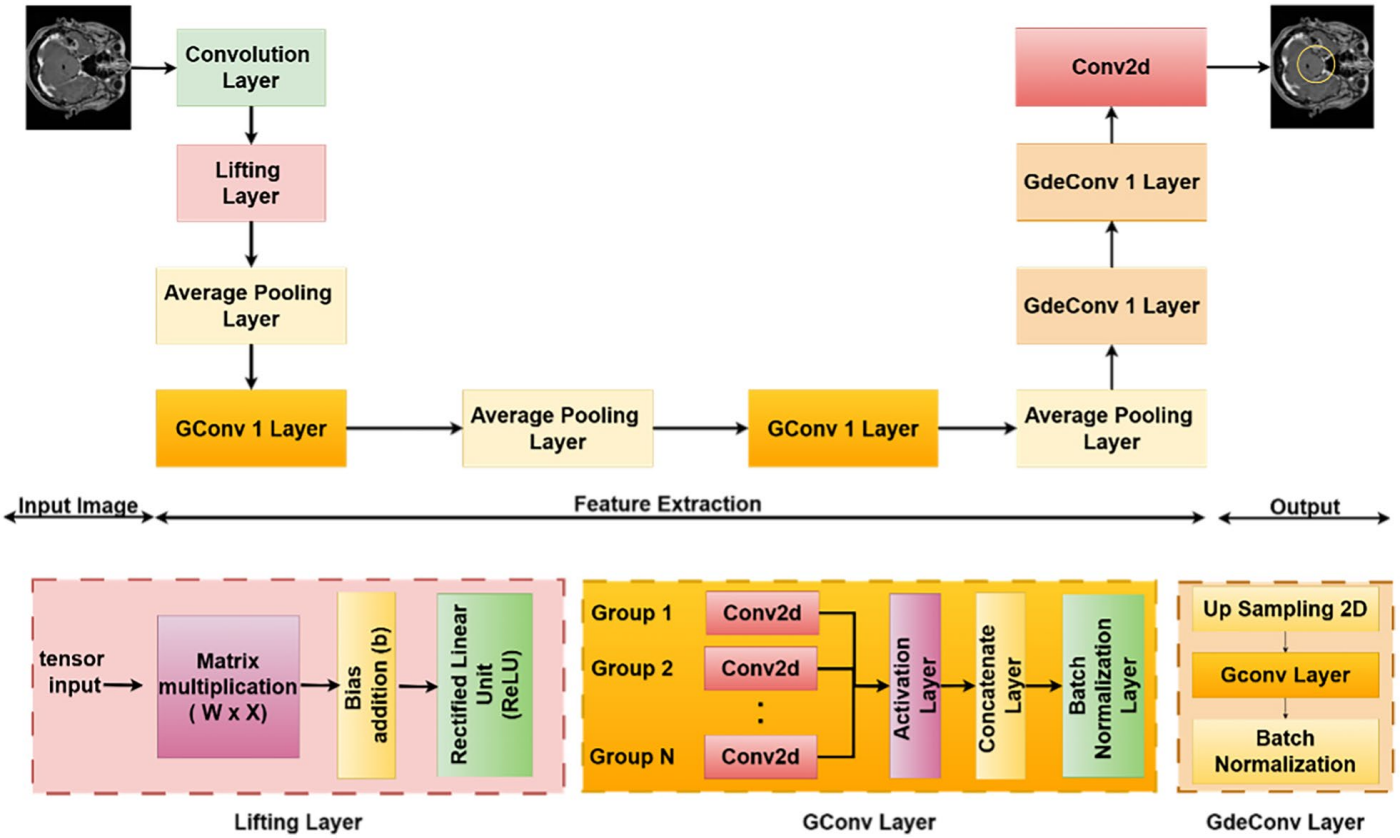
Mod-Seg-SE(2) architecture for brain tumor segmentation. The network includes an initial convolution and lifting layer, group convolutions for transformation robustness, and group deconvolutions (GdeConv) in the decoder for precise segmentation mask reconstruction

For the segmentation task, the Mod-Seg-SE(2) uses a U-Net-inspired structure, where the encoder is the same as the previously described classification network. Figure 3 shows the architecture of the Mod-Seg-SE(2) CNN. The model includes the same lifting layer and stack of group convolution layers, enabling it to extract orientation-aware features that are equivariant to both rotation and translation (see Section 2: Mod-Seg-SE(2) Architecture in Supplementary File 1). Reusing this encoder allows the segmentation branch to benefit from a robust and geometrically informed feature representation that is already optimized for tumor classification.

The decoder replaces standard deconvolution layers with group deconvolutions (GDeconv), which perform transposed convolutions over the SE(2) group. Unlike traditional upsampling layers, which operate only in the spatial domain, GDeconv layers reconstruct features in both spatial and orientation dimensions. This allows the decoder to upsample SE(2)-structured feature maps while maintaining their equivariant properties. Consequently, the segmentation outputs preserve the model’s geometric consistency and remain stable under transformations such as rotation and translation. Pairing a shared classification encoder with an equivariant decoder in this architectural design ensures that Mod-SE(2) can produce accurate and consistent segmentation masks across a wide range of tumor presentations and orientations. This structure enables the network to handle spatial variability in medical images without relying on extensive data augmentation, making it highly effective for brain MRI segmentation.

**Algorithm 1:**
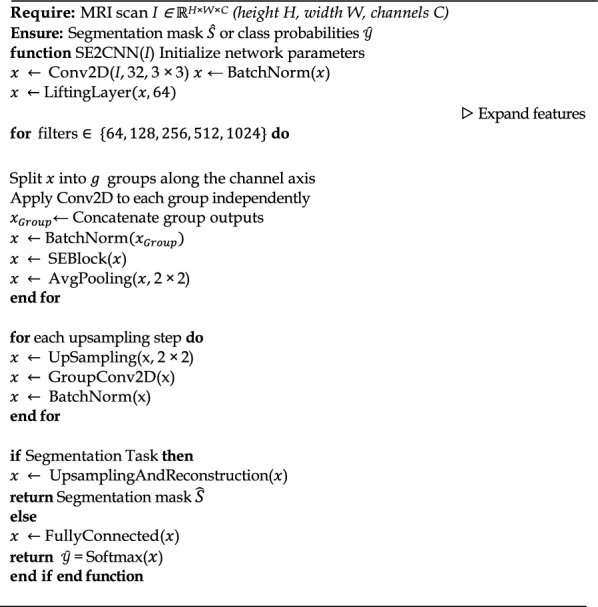
Mod-SE(2) architecture

#### Dataset and preprocessing

We evaluated Mod-SE(2) using three MRI datasets and two medical image datasets, which covered both classification and segmentation tasks (see Section 3: Dataset and preprocessing in Supplementary File 1). The datasets used in our experiments are summarized in Table 1. The MRI Alzheimer’s dataset is primarily used for classification tasks, distinguishing between different stages of Alzheimer’s disease. The Public MRI Brain Tumors dataset contains labeled tumor images for classification and segmentation tasks. It is labeled as public because the dataset is available publicly on the Kaggle platform. The private MRI Brain Tumors dataset was obtained from National Taiwan University (NTU) Hospital, Taiwan, and is not publicly available online (see Supplementary File 2-5). These two datasets consist of high-resolution tumor scans and provide an additional test set to evaluate our model’s generalizability. The skin lesion and blood cell datasets are used for segmentation tasks. They consist of paired images and corresponding ground truth masks that enable the model to accurately detect and localize target objects, such as lesions or cells, within their surrounding context. For the Skin Lesion dataset, the lesion types include malignant melanoma, benign melanocytic nevus, and seborrheic keratosis. For the Blood Cell dataset, the annotated white blood cell types include lymphocytes, monocytes, eosinophils, and neutrophils. These datasets were included solely to evaluate the generalizability of Mod-Seg-SE(2) to other medical image segmentation domains beyond brain MRI.

**Table 1:** Overview of MRI datasets used for evaluation

Dataset	Type	Train	Test
BraTS2020 [38]	Segmentation	628	110
Skin Lesion [39]	Segmentation	900	100
Blood Cell [40]	Segmentation	1169	159
MRI Brain Tumors (Public) [41]	Classification	2235	496
MRI Brain Tumors (Private)	Classification & segmentation	3878	969

**Fig. 4:**
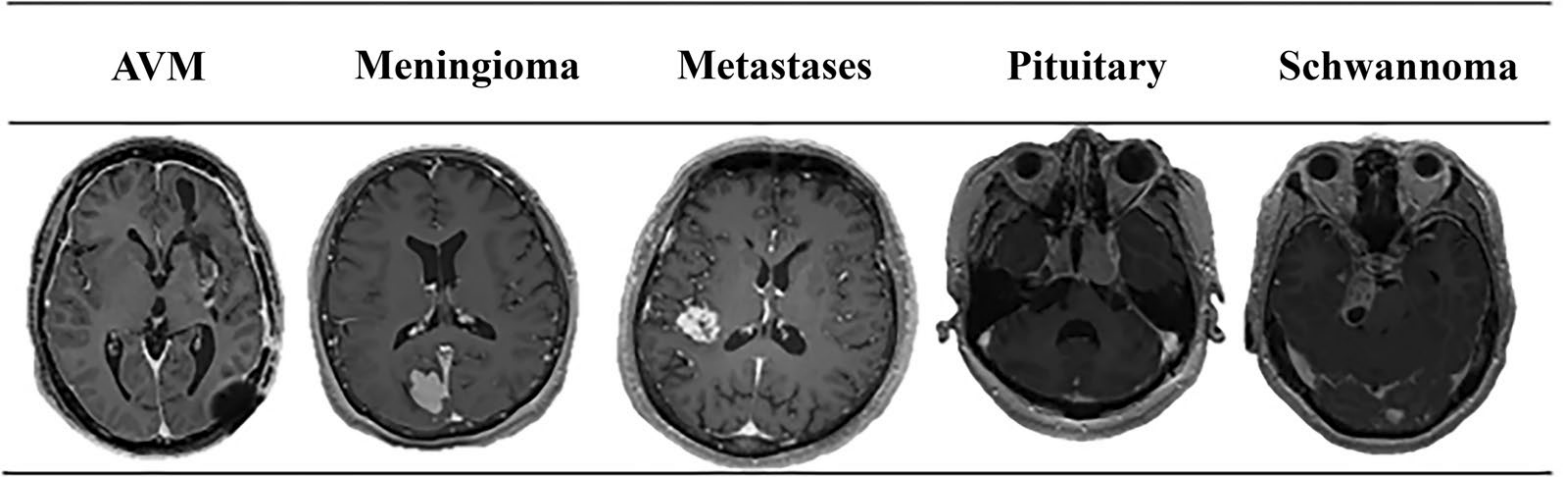
Representative samples from the Private MRI Brain Tumor dataset collected from NTU Hospital, Taiwan. The images illustrate the diversity in tumor size, location, and morphology (see Supplementary File 2–5)

Figure 4 shows representative examples from our private MRI brain tumor dataset under real-world clinical settings. Experienced radiologists have annotated each scan to serve as ground truth for classification and segmentation tasks. The examples demonstrate the significant variability in tumor size, shape, intensity, and anatomical location, which reflects the inherent heterogeneity encountered in this field.

To ensure consistency across datasets and improve model performance, we applied a standardized preprocessing pipeline before training. This pipeline included standardization to uniform resolution, histogram equalization, data augmentation, and lastly, pixel value normalization. First, we resized all MRI scans to a uniform resolution of 256×256 pixels to maintain consistency across datasets and facilitate batch processing in deep learning models. Then, we performed intensity normalization using histogram equalization to enhance contrast and improve tumor visibility. This mitigates variations in brightness and contrast caused by different MRI acquisition protocols. To increase the dataset’s diversity and improve the model’s robustness to rotational features, minor augmentations were applied to the training data, including random rotations (up to ±15°) and scaling (±10%). These transformations help the model generalize better to unseen orientations and variations in tumor shape. Finally, all the image pixel intensities were normalized to the [0,1] range to stabilize training and prevent numerical instability. Normalization ensures that input values remain within a consistent range, thereby optimizing convergence speed during training. These preprocessing steps ensure that the input data remains well-conditioned for deep learning models, which minimizes the impact of variability across MRI scans and improves Mod-SE(2)’s overall generalizability in brain tumor and Alzheimer’s disease classification.

#### Training strategy and hyperparameter selection

For classification, three MRI datasets are used, which are the MRI Alzheimer’s dataset for four-class classification (Mild Demented, Moderate Demented, Non-Demented, Very Mild Demented); the public MRI Brain Tumor dataset; and the private dataset for multi-class tumor classification, assessing the models’ ability to differentiate pathological conditions.

For segmentation, the datasets include blood cell segmentation, skin lesion segmentation, and the private MRI brain tumor segmentation dataset across five tumor classes: AVM, Meningioma, Pituitary, Metastases, and Schwannoma. The segmentation models addressed challenges such as cell overlap, boundary variations, and pixel-wise tumor delineation. Unlike standard CNNs that require extensive augmentation, Mod-SE(2) is inherently invariant to transformations, thus reducing the need for augmentation. The model is trained using the following configuration, as summarized in Table 2.

**Table 2:** Training Hyperparameters and Justifications

Parameter	Value	Justification
Optimizer	Adam	Adaptive learning for stable convergence
Learning rate	0.001	Tuned via validation set performance
Batch size	8 (Classification), 32 (Segmentation)	Memory-efficient training
_Loss function_	Categorical Cross-Entropy (Classifications) Jaccard Loss (Segmentation)	Better for class imbalance
_Training duration_	100 epochs (Classification), 70 epochs (Segmentation)	Optimal convergence point

We ran all experiments on an NVIDIA GeForce RTX 3090, using TensorFlow 2.4 and CUDA 11. This setup enabled efficient training of the data and computationally intensive group convolutions. We applied the following regularization techniques were applied: batch normalization stabilizes training by normalizing activations across layers, dropout (0.4 probability) reduces overfitting by randomly deactivating neurons during training, and weight decay (0.0005) acts as a regularization term to prevent excessive weight magnitudes. These techniques collectively improve model robustness, ensuring stable training and better generalization to unseen MRI scans.

### Evaluation metrics

We evaluate the performance of the Mod-SE(2) CNN in classification (Mod-Cls-SE(2)) and segmentation (Mod-Seg-SE(2)) tasks using various metrics. We measure classification tasks using accuracy, precision, recall, and F1 score. The segmentation task is evaluated based on its dice coefficient, Intersection over Union (IoU), precision, and recall value. These metrics provide a comprehensive evaluation of the effectiveness of classification, which is crucial in medical imaging, where false positives and false negatives can have significant clinical consequences.

#### Classification metrics

Accuracy ($Acc$) is a measure of the model's overall correctness. It is calculated by dividing the number of correctly classified instances by the total number of instances:

8 $$Acc= \frac{True\,Positives + True\,Negatives}{Total\,samples}$$

This metric provides an overview of the model’s performs but may not sufficient in cases of class imbalance.

Precision (${P}_{c}$) quantifies the proportion of correctly predicted positive cases among all positive predictions. It ensures that the model minimizes false alarms, which is crucial in medical diagnostics. Similarly, recall (${R}_{c}$), also known as sensitivity, measures the model’s ability to detect actual positive cases, ensuring that malignant cases are not overlooked. These metrics are defined as follows:

9 $${P}_{c}= \frac{True\,Positives }{True\,Positives + False\,Positive}$$

10 $${R}_{c}= \frac{True\,Positives }{True\,Positives + False\,Negatives}$$

Finally, the F1 score ($F1$) provides a balanced measure of precision and recall by computing their harmonic mean. This metric is particularly useful in imbalanced datasets, where a high precision or recall alone may not be sufficient. It is defined as:

11 $$F1 = 2\frac{{P_{c} \cdot R_{c} }}{{P_{c} + R_{c} }}$$

This equation illustrates the trade-off between precision and recall, ensuring optimal classification performance, particularly in situations where false positives and false negatives carry significant consequences. Together, these metrics provide a comprehensive evaluation of the Mod-Cls-SE(2)’s classification performance. High values, particularly for the $F1$ score, demonstrate the model’s ability to accurately detect true positives while minimizing false positives and false negatives, making the model highly effective for real-world medical imaging applications.

#### Segmentation metrics

The Dice coefficient (D) measures the overlap between predicted segmentation and the ground truth. It provides a score that reflects how closely the model’s output matches the actual tumor regions. It is defined as follows:

12 $$D= \frac{2 \dot | A \cap B |}{\left|A\right|+|B|}$$

where A is the predicted segmentation and B is the ground truth.

The Intersection over Union (IoU) evaluates the ratio of the intersection to the union of the predicted and true regions, representing segmentation accuracy:

13 $$IoU= \frac{|A\cap B|}{|A\cup B|}$$

The Precision (${P}_{s}$) quantifies the proportion of correctly predicted positive pixels among all pixels predicted as positive. It is defined as:

14 $${P}_{s}= \frac{True\,Positives }{True\,Positives + False\,Positive}$$

The Recall (${R}_{s}$), or sensitivity, measures the proportion of actual positive pixels that are correctly identified by the model, ensuring minimal false negatives in tumor detection:

15 $${R}_{s}= \frac{True\,Positives }{True\,Positives + False\,Negatives}$$

These metrics assess the Mod-Seg-SE(2)’s ability to segment brain tumors with high accuracy and consistency. The dice coefficient and IoU are key for evaluating spatial overlap, ensuring the model’s clinical viability in medical diagnostics.

## Results

This research evaluated segmentation and classification tasks using various medical imaging datasets, including blood cell images, skin lesion images, brain MRI scans (both public and private), as well as MRI data from patients with Alzheimer's disease. All images were standardized to a 2D format and underwent normalization to ensure consistency in pixel intensity and reduce variations from acquisition conditions [42].

The Mod-SE(2) modification was compared with VGG16 [43], VGG19 [44], ResNet50 [45], ResNet101 [46], HoverNet [47], and Harmonic Net [18], as well as U-Net based segmentation model from the work of [12, 48] and No New U-Net (NN U-Net) [49], ensuring a comprehensive evaluation against both traditional deep learning and specialized segmentation networks. Using roto-translation equivariance, Mod-SE(2) demonstrated improved spatial consistency and reduced false positives, validating its robustness in multiple medical imaging tasks.

### Classification performance

Table 3 shows the classification performance across the model. We compared the predicted labels generated by Mod-Cls-SE(2) with the expert-annotated ground truth labels provided in each dataset in public MRI brain classification. It achieves the highest accuracy of 0.906, precision of 0.910, recall of 0.904, and an exceptional F1 score of 0.908. These results highlight the model’s robustness in handling spatial transformations and its ability to maintain both sensitivity and specificity without relying excessively on data augmentation. In contrast, HarmonicNet achieves a higher recall of 0.875 compared to most conventional models, although it slightly struggles with a precision of 0.830, resulting in a balanced F1 score of 0.852. Traditional CNN-based models such as VGG16, VGG19, and ResNet variants exhibit significantly lower performance. Their accuracy values range from 0.755 to 0.8, which reinforces their limitations in dealing with spatial variability. VGG16 shows moderate recall of 0.795 and slightly better precision of 0.805, indicating a tendency toward false negatives. These results confirm that the roto-translation equivariance of Mod-SE(2) effectively enhances feature stability, making it a more reliable and computationally efficient solution for brain MRI classification. Remarkably, despite its more sophisticated and computationally complex architecture, Mod-Cls-SE(2) has fewer parameters and is more efficient than denser models, such as VGG19 and ResNet101, in terms of computational resource utilization and performance. This highlights its efficiency and effectiveness in clinical MRI classification.

**Table 3:** Performance metrics for public MRI brain classification, comparing Mod-Cls-SE(2), HoverNet, HarmonicNet, VGG16, VGG19, ResNet50, and ResNet101

Model’s type	Model’s name	Accuracy	Precision	Recall	F1 Score
^Group− equivariant network^	^Mod−Cls−SE(2)^	^0.906^	^0.910^	^0.904^	^0.908^
	^HarmonicNet^	^0.863^	^0.830^	^0.875^	^0.852^
Non group- equivariant network	^HoverNet^	^0.780^	^0.790^	^0.770^	^0.780^
	^VGG16^	^0.800^	^0.805^	^0.795^	^0.800^
	^VGG19^	^0.763^	^0.771^	^0.753^	^0.762^
	^ResNet50^	^0.755^	^0.760^	^0.740^	^0.750^
	^ResNet101^	^0.771^	^0.783^	^0.763^	^0.772^

Table 4 shows the most effective model for private MRI brain classification. It achieves the highest performance across all evaluation metrics, with an accuracy of 0.931, precision of 0.947, recall of 0.918, and F1 score of 0.933. This comprehensive superiority highlights the Mod-SE(2) based model’s robustness in correctly identifying positive cases while maintaining low false positive and false negative rates. Compared to all other models, including HarmonicNet, HoverNet, VGG variants, and ResNet architectures, Mod-Cls-SE(2) outperforms them all in every aspect. HarmonicNet, while also a group-equivariant model, trails behind in all metrics. Traditional CNN-based models such as VGG16, VGG19, ResNet50, and ResNet101 show notably lower performance, struggling particularly with recall and F1 score, which are key indicators of balanced and reliable classification. These findings underscore the value of group-equivariant architectures, particularly Mod-SE(2)’s built-in roto-translation equivariance, in enhancing generalization and spatial understanding. This makes Mod-Cls-SE(2) especially well-suited for complex medical imaging tasks, such as brain MRI classification.

**Table 4:** Performance metrics for private MRI brain classification, comparing Mod-Cls-SE(2), HoverNet, HarmonicNet, VGG16, VGG19, ResNet50, and ResNet101

Model’s type	Model’s name	Accuracy	Precision	Recall	F1 Score
Group- equivariant network	Mod-Cls-SE(2)	0.931	0.947	0.918	0.933
	HarmonicNet	0.885	0.814	0.896	0.853
Non group- equivariant network	HoverNet	0.784	0.863	0.717	0.783
	VGG16	0.837	0.904	0.760	0.826
	VGG19	0.730	0.859	0.576	0.690
	ResNet50	0.440	0.766	0.149	0.249
	ResNet101	0.788	0.883	0.682	0.770

Table 5 shows Mod-Cls-SE(2)’s performance across five binary tumor classification tasks and highlights its ability to adapt to different tumor types with varying levels of sensitivity and specificity.

**Table 5:** Performance of Mod-SE(2) in five binary classification tasks, demonstrating its robustness in brain tumor detection

No	Classification task	Accuracy	Precision	Recall	F1 Score
1	AVM vs non AVM	0.923	0.945	0.905	0.924
2	Meningioma vs non meningioma	0.931	0.952	0.914	0.932
3	Pituitary vs non pituitary	0.935	0.960	0.920	0.939
4	Metastases vs non metastases	0.925	0.940	0.905	0.923
5	Schwannoma vs non schwannoma	0.928	0.945	0.910	0.928

Among the tumor types, the Pituitary task achieves the highest precision of 0.960 and recall of 0.920, resulting in the best F1 score of 0.939. This indicates that Mod-Cls-SE(2) is especially effective at identifying pituitary tumors with both high sensitivity and specificity. Meningioma also shows a balanced performance with a precision of 0.952, recall of 0.914, and F1 score of 0.932, while Schwannoma demonstrates a similarly robust performance precision of 0.945, recall of 0.910, and F1 score of 0.928. In contrast, AVM vs Non AVM and Metastases, though slightly lower, still maintain high overall performance with precision of 0.940 – 0.945, recall of 0.905, and F1 scores of 0.924 and 0.923, respectively. These results collectively suggest that Mod-Cls-SE(2) consistently balances precision and recall across different tumor classifications and excels in scenarios where high sensitivity is critical.

Table 6 shows that Mod-Cls-SE(2) outperforms traditional models, including VGG16, VGG19, ResNet50, and ResNet101, across all metrics. It achieved the highest accuracy of 0.914, far surpassing ResNet101’s 0.682. This demonstrates Mod-Cls-SE(2)’s superior capability in brain tumor classification. This improvement highlights the effectiveness of its roto-translation invariance in handling spatial and rotational variations without extensive data augmentation. Furthermore, Mod-Cls-SE(2) consistently delivered higher precision of 0.923, recall of 0.909, and F1 score of 0.916 than the conventional CNN-based models. Mod-Cls-SE(2) also demonstrated a reduction in inference time during the evaluation phase, making it suitable for real-time clinical applications. The model’s computational efficiency was also a significant advantage, requiring only 2.5 hours for training compared to 6–7 hours for the ResNet-based models. With considerably shorter inference and training time, Mod-Cls-SE(2) not only reduces computational load but also outperforms existing CNN-based models in classification tasks. Overall, these results underscore how the inherent geometric priors of the Mod-SE(2) based model, especially Mod-Cls-SE(2), contribute to both improved performance and reduced computational cost.

**Table 6:** Average performance comparison of Mod-Cls-SE(2), VGG16, VGG19, ResNet50, and ResNet101. Mod-Cls-SE(2) achieves the best overall performance and fastest computation time, demonstrating its efficiency in medical image classification

Model’s type	Model’s name	Accuracy	Precision	Recall	F1 score	Time
Group- equivariant network	Mod-Cls-SE(2)	0.914	0.923	0.909	0.916	2.5 h
Non group- equivariant network	VGG16	0.705	0.688	0.676	0.677	6 h
	VGG19	0.635	0.607	0.582	0.567	5.5 h
	ResNet50	0.548	0.562	0.422	0.402	6.2 h
	ResNet101	0.682	0.659	0.597	0.623	7 h

In addition to evaluating accuracy and training efficiency, we assessed the model’s suitability for real-time clinical applications by evaluating the inference time per image. Table 7 shows that the proposed Mod-Cls-SE(2) achieves a remarkably low average inference time of 0.1099 seconds per image. This is significantly faster than all baseline models, including VGG16 with a time of 0.2242 seconds, VGG19 of 0.8326 seconds, ResNet50 of 10.2734 seconds, and ResNet101 of 10.3045 seconds. This substantial difference highlights the computational efficiency of Mod-Cls-SE(2), which reduces the need for the excessive feature computations typically required by deeper, conventional CNNs. The combination of this speed advantage, combined with high classification accuracy, reinforces the potential of Mod-Cls-SE(2) for deployment in time-sensitive environments, such as clinical decision support systems and point-of-care diagnostics.

**Table 7:** Average inference time per image for each model

Model’s name	Inference (seconds/image)
Mod-Cls-SE(2)	0.1099
VGG16	0.2242
VGG19	0.8326
ResNet50	10.2734
ResNet101	10.3045

**Fig. 5:**
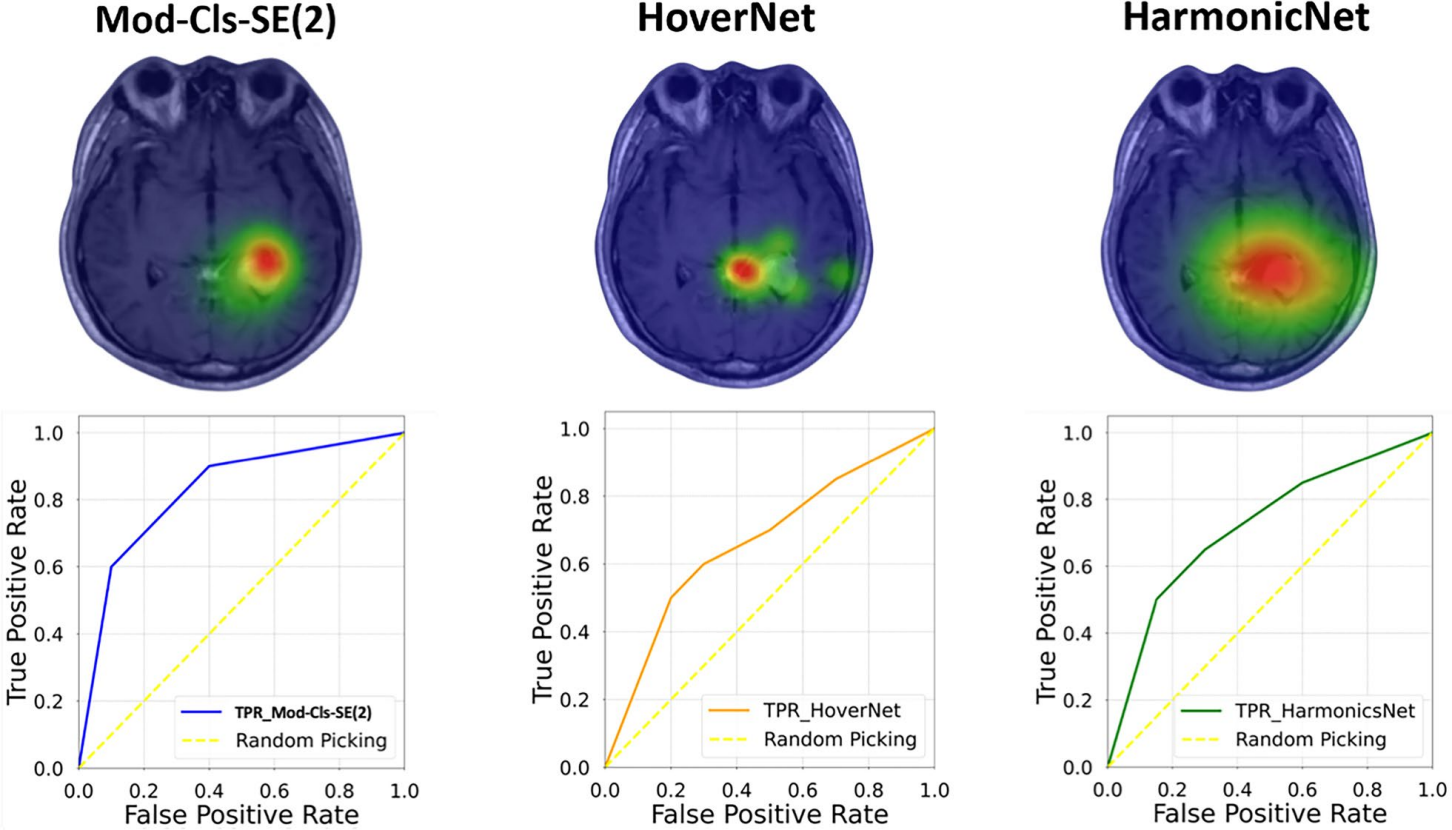
Heatmap and ROC curves comparison for brain tumor classification using three rotational models. A larger AUC indicates better classification performance, minimizing false positives while maximizing true positives. **a** Mod-Cls-SE(2) demonstrates the most accurate and focused segmentation, with a concentrated heatmap around the tumor region. The corresponding ROC curve (blue) indicates high classification performance with a large area under the curve (AUC), benefiting from its roto-translation equivariant architecture and reduced reliance on data augmentation. **b** HoverNet exhibits a more diffuse heatmap, indicating less precise feature localization. The associated ROC curve (orange) shows increased false positives, reflecting limitations in handling rotational variance despite the use of post-processing techniques. **c** HarmonicNet yields the least accurate segmentation, with a broad and less informative heatmap. Its ROC curve (green) is attributed to its partial rotation equivariance via circular harmonics without translation invariance

Overall, the model performs well, but errors between neighboring severity levels suggest room for improvement. Enhancements such as data augmentation, threshold adjustments, or network modifications could help reduce misclassifications. Figures 5a-c show a heatmap area comparison and ROC curve for three brain tumor classifications in the Mod-Cls-SE(2) rotational model, HoverNet, and HarmonicNet, respectively, using a sample MRI scan. The heatmap shows how each rotational model highlights important features from the dataset. In Figure 5a, the Mod-Cls-SE(2) model demonstrates the most accurate and focused segmentation, with its heatmap showing a highly concentrated prediction of the tumor. The associated TPR and FPR graph indicates superior performance, benefiting from its unified geometric framework that ensures both rotation and translation equivariance, requiring less data augmentation. In contrast, Figure 5b shows that HoverNet produces a less precise segmentation with a more spread-out heatmap, and the graph shows more false positives, reflecting its limitation in handling variations in orientation. Although it uses post-processing techniques, it lacks full roto-translation invariance. Figure 5c shows that HarmonicNet produces the least accurate segmentation. Its heatmap is diffuse, and its performance is the lowest. This is because HarmonicNet only addresses rotation equivariance through circular harmonics without fully accounting for translation. These results prove that the Mod-Cls-SE(2) outperforms both HoverNet and HarmonicNet, particularly in tasks requiring the handling of high spatial variability, offering more robust and precise segmentation.

### Segmentation performance

The selection of baseline models is based on their proven effectiveness in deep learning tasks. VGG and ResNet are included because of their strong ability to extract hierarchical features, making them standard choices for learning features in convolutional neural networks. However, these architectures lack inherent geometric awareness, limiting their ability to capture spatial transformations such as rotation and translation [50]. U-Net and NN U-Net were chosen because they are state-of-the-art architectures for image segmentation, especially in medical imaging and remote sensing applications. Their robust segmentation performance allows for a fair comparison when evaluating the effectiveness of the proposed approach [13]. However, these models do not explicitly incorporate mechanisms for handling geometric transformations, which are crucial for tasks requiring spatial equivariance. To assess the impact of incorporating geometric priors further, we compared Mod-Seg-SE(2) with the baseline architectures.

We quantitatively evaluated segmentation performance by comparing model-generated masks with expert-annotated ground truth masks. For the MRI brain tumor datasets, certified radiologists delineated ground truth annotations based on clinical diagnostic protocols, ensuring high spatial fidelity and anatomical accuracy. For the skin lesion dataset, the ground truth segmentation was established using clinically validated methods, which are follow-up examinations, expert consensus, and confirmation via in vivo confocal microscopy, as detailed in the dataset's metadata. For the blood cell dataset, ground truth masks for white blood cells were created through manual pixel-wise annotations by trained experts using stained microscopic images, ensuring precise delineation of cellular boundaries. We use the Skin Lesion and Blood Cell segmentation experiments are secondary, cross-domain validation studies. They are intended to demonstrate the model’s ability to generalize to diverse medical imaging modalities. We compared the segmentation performance of Mod-Seg-SE(2), U-Net, and NN U-Net using the Dice coefficient, precision, recall, intersection over union (IoU), and F1 score. These metrics evaluate tumor boundary delineation, false positive suppression, and alignment with ground truth.

Table 8 summarizes the comparative results across these key metrics. The Mod-Seg-SE(2) demonstrated superior segmentation performance in MRI Brain Tumors (Private) , achieving the highest dice score of 0.635 and IoU value of 0.715. These scores surpassed those of U-Net with 0.375 and 0.465 in dice score and IoU, respectively, and NN U-Net with 0.505 and 0.595 in dice score and IoU, respectively. The Mod-Seg-SE(2) also achieved a higher precision of 0.685 and recall of 0.655, indicating a balanced ability to correctly identify tumor regions while minimizing both false positives and false negatives. In contrast, NN U-Net exhibited a lower recall of 0.545, which could cause the model to miss tumor regions. This is a critical issue in clinical diagnosis. The incorporation of roto-translation equivariance in Mod-Seg-SE(2) contributes to its robust boundary delineation and spatial consistency. The performance of Mod-Seg-SE(2) is also proven by its high performance by achieving 0.9503 and 0.9616 in dice score and IoU, respectively. In contrast, both U-Net (dice score of 0.797 and IoU of 0.742) and NN U-Net (dice score of 0.815 and IoU of 0.802) yield notably lower scores, which are critical for accurate tumor segmentation. These evaluations were conducted on the BraTS2020 dataset, a well-established and widely used benchmark for brain tumor segmentation tasks. The consistent superiority across multiple evaluation metrics underscores the framework’s reliability and effectiveness and makes the model well-suited for brain tumor segmentation in medical imaging applications. On the Skin Lesion dataset, Mod-Seg-SE(2) achieved the highest performance with a dice score of 0.900 and IoU of 0.8601, surpassing U-Net and NN U-Net. Its high precision of 0.9345 and recall of 0.8810 demonstrate its ability to detect boundaries in dermatological segmentation. The model outperformed others with a Dice score of 0.8788 and an IoU of 0.7851. Its precision of 0.8982 and recall of 0.8223 indicate its robustness in handling overlapping cells. These results confirm that Mod-Seg-SE(2) generalizes effectively across different medical imaging tasks.

**Table 8:** Performance comparison of segmentation models on medical imaging datasets. Mod-Seg-SE(2) surpasses U-Net and NN U-Net in dice score, IoU, Precision, and Recall

Dataset	Model	Group equivariant network	Dice score	IoU	Precision	Recall
Blood cell	Mod-Seg-SE(2)	✓	0.8788	0.7851	0.8982	0.8223
Blood cell	U-Net		0.6983	0.6884	0.7082	0.6831
Blood cell	NN U-Net		0.7518	0.7248	0.8709	0.7307
Skin lesion	Mod-Seg-SE(2)	✓	0.9000	0.8601	0.9345	0.8810
Skin lesion	U-Net		0.8060	0.7311	0.8476	0.8145
Skin lesion	NN U-Net		0.8420	0.8345	0.8798	0.8309
MRI brain	Mod-Seg-SE(2)	✓	0.635	0.715	0.685	0.655
MRI brain	U-Net		0.375	0.465	0.465	0.465
MRI brain	NN U-Net		0.505	0.595	0.505	0.545
BraTS2020	Mod-Seg-SE(2)	✓	0.9503	0.9616	0.947	0.930
BraTS2020	U-Net		0.797	0.742	0.715	0.762
BraTS2020	NN U-Net		0.815	0.802	0.805	0.802

**Fig. 6:**
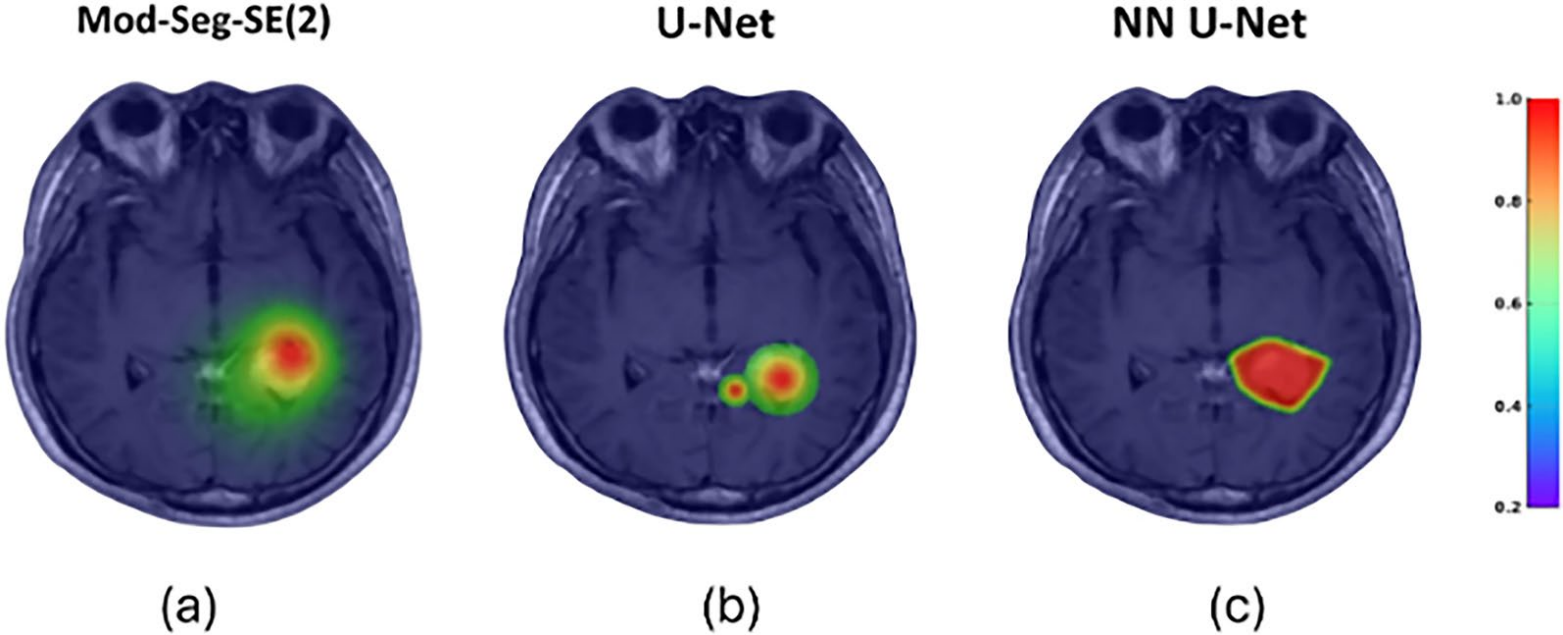
Heatmap visualization of predicted tumor regions in an MRI scan. Colored heatmap overlays reflect each model’s confidence in tumor localization, with Mod-Seg-SE(2) demonstrating the most balanced and reliable performance across both completeness and spatial specificity. **a** Mod-Seg-SE(2) achieves the most accurate and spatially precise segmentation, with sharply defined tumor boundaries and high confidence in localized predictions. **b** U-Net produces a more localized segmentation that is relatively limited in scope, potentially missing peripheral tumor regions. **c** NN U-Net yields a broader segmentation, capturing more of the tumor area but with reduced boundary precision

Figure 6a-c compares brain tumor segmentation results using three deep learning models: Mod-Seg-SE(2), U-Net, and NN U-Net, respectively. Figure 6a shows that Mod-Seg-SE(2) produces clearer and more accurate segmentation. In contrast, Figure 6b shows that U-Net provides more localized segmentation that may lack comprehensiveness. In contrast, NN U-Net produces a broader segmentation that appears less precise in Figure 6c, but effectively captures the tumor region. Overall, Mod-Seg-SE(2) appears to offer the most optimal segmentation performance of the three models.

**Fig. 7:**
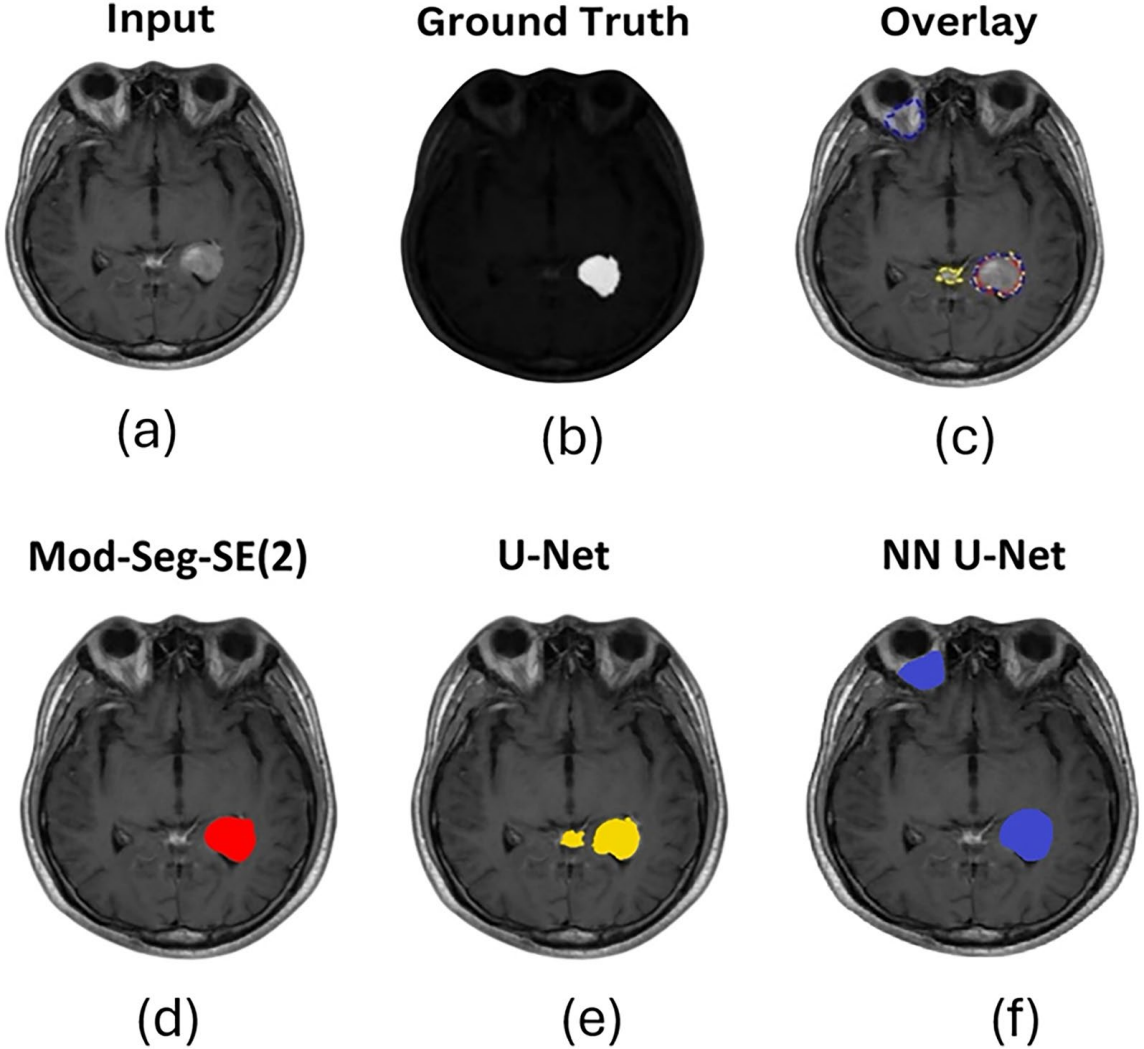
Comparison of brain tumor segmentation results using Mod-Seg-SE(2), U-Net, and NN U-Net. The input MRI scan and corresponding ground truth mask are provided for reference. This visual comparison highlights the superior spatial feature capture of Mod-Seg-SE(2), while NN U-Net offers competitive performance in delineating tumor boundaries. **a** Input MRI scan. **b** Ground truth segmentation mask for reference. **c** Overlay of predicted segmentation results from Mod-Seg-SE(2), U-Net, and NN U-Net, highlighting areas of overlap and divergence across models. **d** Output from Mod-Seg-SE(2), showing high spatial accuracy and strong alignment with the ground truth. **e** Output from U-Net, demonstrating more localized segmentation with some under-segmentation. **f** Output from NN U-Net, providing broader coverage with relatively accurate boundary definition

Figure 7 provides a qualitative comparison of segmentation results across several methods to further demonstrate the effectiveness of Mod-Seg-SE(2) compared to existing segmentation models. Figure 7a shows the input MRI scan, and Figure 7b shows the corresponding ground truth. Figure 7c shows an overlay of the predicted segmentation outputs from Mod-Seg-SE(2), U-Net, and NN U-Net. This overlay offers a visual summary of the method’s relative performance and highlighting areas of agreement and discrepancy. Figures 7d to 7f show the individual outputs from Mod-Seg-SE(2), U-Net, and NN U-Net, respectively. This comparison emphasizes Mod-Seg-SE(2)’s ability to capture spatial features, while NN U-Net offers competitive boundary precision.

**Fig. 8:**
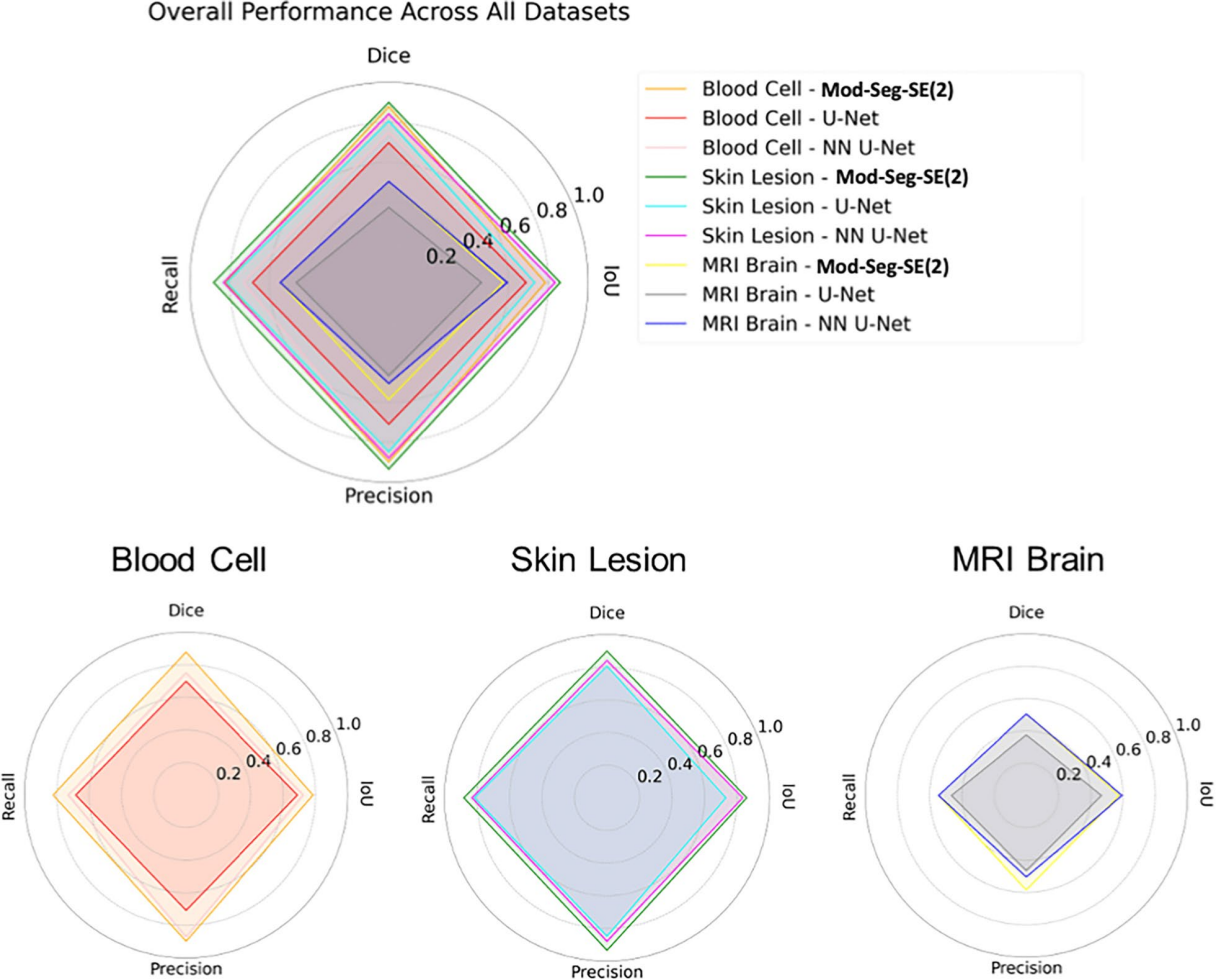
Radar chart comparison of Mod-Seg-SE(2), U-Net, and NN U-Net across Blood Cell, Skin Lesion, and MRI Brain datasets, demonstrating the superior performance of Mod-SE(2) in key segmentation metrics

Figure 8 shows a radar chart that compares the performance of segmentation across multiple modalities, such as blood cell, skin lesion, and MRI brain datasets. The results demonstrate that the Mod-SE(2) based model outperforms U-Net and NN U-Net in terms of dice score, IoU, precision, and recall, highlighting its superior segmentation accuracy. Importantly, the model’s strong performance across different datasets showcases its generalizability and robustness. It proves its capability to handle diverse medical imaging modalities beyond just MRI, making it a versatile tool for various segmentation tasks.

### Ablation study

To determine which Mod-SE(2) components contribute most to performance gains, we conducted an ablation study. We systematically removed elements such as roto-translation equivariance, feature recalibration layers (SE-Blocks), and group convolutions, and performed validations using the MNIST dataset [51]. On observing classification model performance and the COCO dataset [52], and on observing segmentation model performance. The results of this study, summarized in Table 9, confirm that the Mod-SE(2) model consistently outshines all ablated versions, achieving the highest classification accuracy of 0.9899 and a segmentation dice score of 0.9514. The integration of roto-translation equivariance and feature recalibration ensures that the model can robustly generalize across different spatial orientations and feature variations, leading to improved feature representation and decision-making. This happened because for a standard convolutional layer:

**Table 9:** Ablation study results for Mod-SE(2)

Model variant	Parameters	Classification accuracy	Segmentation dice score
FullMod-SE(2) (Proposed)	12,284,518	0.9899	0.9514
Without Roto-Translation	16,741,300	0.9839	0.9450
Without Feature Recalibration	12,284,518	0.9773	0.9421
Without Group Convolution	16,741,300	0.9875	0.9498

16 $$\text{Parameters }= \left(\text{Kernel Height }\times \text{ Kernel Width }\times \text{ Input Channels}\right)\times \text{ Output Channels }+\text{ Bias Parameters}$$

Meanwhile, for a group convolutional layer:

17 $$\text{Parameters }= \left(\text{Kernel Height }\times \text{ Kernel Width }\times \frac{\text{Input Channels}}{\text{Groups}}\right) \times \frac{\text{Output Channels}}{\text{Groups}}+\text{Bias Parameter}$$

Group convolutions divide channels into smaller groups, each of which processes a fraction of the input channels. This reduces the number of trainable parameters per layer. The model without roto-translation equivariance experiences an increase in the number of parameters. This occurs because the group convolutions, which preserve roto-translation equivariance, are replaced with standard convolutions that process all input channels simultaneously. Each layer now has significantly more parameters because instead of dividing channels into groups, the full input feature map is processed at every layer. Similarly to models without rotational translation, the model without group convolutions replaces all group convolutions and replaces them with standard convolutions. Standard convolutions have much larger parameters because they require more filters per layer, which increases the model’s memory and computational cost.

## Discussion

Accurate brain tumor segmentation is crucial for diagnosis and treatment planning in medical imaging. Although traditional CNN-based segmentation models, such as U-Net and NN U-Net, have demonstrated strong performance in delineating tumor regions, they struggle with spatial inconsistencies caused by variations in orientation, translation, and acquisition conditions. The implementation of Mod-SE(2) in medical imaging represents a significant advancement in AI-driven radiology. Unlike traditional CNNs, which require manual data augmentation to address spatial transformations, Mod-SE(2) inherently encodes roto-translation invariance. This allows it to adapt to variations in image orientation. This ensures consistent feature extraction across different MRI acquisition angles, reducing the variability that often affects traditional deep learning models. Integrating geometric priors directly into the network architecture, Mod-SE(2) enhances segmentation accuracy, minimizing the risk of false positives and false negatives in brain tumor detection. Furthermore, Mod-SE(2) demonstrated the ability to detect features as small as 5×5 pixels, equivalent to approximately 2.5 mm in the synthetic dataset. This highlights its high spatial sensitivity and suitability for recognizing fine-scale tumor recognition (see Section 4: Fine-scale feature detection in Supplementary File 1). Additionally, its optimized design leads to faster inference times, making it well-suited for real-time clinical workflows. This combination of accuracy, efficiency, and adaptability positions Mod-SE(2) as a highly promising tool for improving AI-assisted diagnostics in neuro-oncology.

With a training time of just 2.5 hours and low computational overhead, Mod-SE(2) is well-suited for deployment in clinical settings. Compared to baseline CNN models, the model demonstrated a noticeable reduction in inference time, highlighting its potential for real-time clinical applications, such as Picture Archiving and Communication Systems (PACS), where speed and accuracy are essential. One of its key applications is in PACS, where Mod-SE(2) can be integrated for real-time MRI analysis. By automating preliminary tumor classification, Mod-SE(2) enables radiologists to streamline their workflow, reducing the time needed for manual review while maintaining diagnostic accuracy. We emphasize that Mod-SE(2) is designed to operate within the imaging stage of the clinical pipeline and is not a substitute for histopathology or molecular analysis. Rather, it can serve as a supportive tool in the interval before histopathology results are available, helping to prioritize cases, assist surgical planning, and potentially accelerate multidisciplinary decision-making. This integration could significantly enhance efficiency in hospitals and imaging centers, enabling faster and more reliable diagnoses. Beyond PACS, Mod-SE(2)’s shorter training time compared to traditional models makes it highly compatible with cloud-based radiology platforms. This efficiency makes it feasible for remote AI-powered diagnostics, supporting telemedicine and decentralized healthcare solutions. These capabilities are valuable in regions with limited access to specialized radiology services, allowing AI-assisted diagnostics to be performed remotely without requiring extensive computational resources. Hence, Mod-SE(2) enhances decision support for radiologists by incorporating feature recalibration mechanisms that generate heatmaps highlighting critical tumor regions. This interpretability feature helps radiologists validate AI predictions, increasing trust and adoption in clinical workflows. By improving both efficiency and transparency, Mod-SE(2) has the potential to become a valuable tool in AI-assisted neuro-oncology and medical imaging. Despite these promising results, this study has certain methodological limitations. Notably, we did not perform k-fold cross-validation due to computational constraints and to maintain a consistent training configuration for direct comparison with baseline models. While this approach facilitated fair benchmarking, we acknowledge that employing k-fold cross-validation could further enhance the statistical rigor and generalizability of our findings. Addressing this limitation will be a focus of future work, aiming to reinforce the robustness of Mod-SE(2) across diverse datasets and clinical environments. Real-world deployment still requires prospective validation in clinical trials to assess its robustness in diverse patient populations and imaging environments. Future work will focus on scaling Mod-SE(2) for larger and broader datasets and multi-institutional studies. The real-time clinical integration, like Monte Carlo simulation for pre-surgical and PET-MRI integration, ensures its reliability and effectiveness in comprehensive practical medical applications. The future work will also extend Mod-SE(2) to Mod-SE(3) using recent advances in spherical harmonic representations and sparse group convolutions [53–57]. This integration will enable volumetric analysis while preserving equivariance across all spatial dimensions. We will focus on balancing computational cost with clinical utility, which remains a key challenge of the implementation.

## Conclusion

We introduce Mod-SE(2), a geometry-aware deep learning framework that improves spatial consistency, interpretability, and efficiency in brain tumor MRI analysis. Mod-SE(2) embeds roto-translational symmetry through lifting layers, group convolutions, and SE blocks. This enables robust, orientation-invariant feature extraction across diverse tumor types and imaging conditions. Extensive experiments demonstrate that Mod-SE(2) significantly improves classification and segmentation performance. It outperforms baseline CNNs in terms of accuracy, precision, and recall, particularly in anatomically complex and rotationally variant cases. The SE(2)-modified model achieved a dice score of 0.90, surpassing the U-Net of 0.863 and outperforming other variants by over 4.5%. For classification, it reached an F1-score of 0.933 on a private MRI dataset, surpassing VGG and ResNet-based models. Mod-SE(2) offers transformative clinical potential for precision medicine by providing spatially consistent, interpretable outputs, which are critical for surgical planning, tumor monitoring, and evaluating treatment responses. Its symmetry-aware design of Mod-SE(2) reduces the reliance on extensive data augmentation by inherently capturing rotational and translational invariances, contributing to accurate tumor boundary delineation. This capability improves decision-making in real-world neuroimaging applications and ensures consistent performance across different MRI acquisition protocols. The model’s spatially precise tumor localization output can be directly utilized as input for Monte Carlo simulations in high-precision preoperative radiotherapy planning, as well as intraoperative surgical guidance. This reduces the risk of incomplete resection or inadvertent damage to critical brain structures. Furthermore, its geometric consistency across modalities facilitates seamless integration with Positron Emission Tomography (PET) imaging, enabling effective fusion of structural and metabolic information. These strengths highlight the versatility of Mod-SE(2) in supporting multimodal imaging pipelines and advanced computational modeling, reinforcing its potential for personalized and comprehensive neuro-oncological care.

## Supplementary Information

Supplementary Material 1

Supplementary Material 2

Supplementary Material 3

Supplementary Material 4

Supplementary Material 5

## Data Availability

The datasets generated and/or analyzed during the current study are available in the Kaggle repository [36–39], but a private dataset is available from the corresponding author on reasonable request.

## Abbreviations

CNN: Convolutional Neural Network
Mod-SE(2): Modified Special Euclidean
Mod-Cls-SE(2): Modified Classification Special Euclidean
Mod-Seg-SE(2): Modified Segmentation Special Euclidean
AVM: Arteriovenous Malformations
MRI: Magnetic Resonance Imaging
GDL: Geometric Deep Learning
G-CNNs: Group Equivariant CNNs
ASD: Autism Spectrum Disorder
AD: Alzheimer Disease
SE: Squeeze-And-Excitation
GdeConv: Group Deconvolutions
IoU: Intersection over Union
