# Supplementary material for: Mod-SE(2): a geometric deep learning framework for brain tumor classification and segmentation in MRI images
Source: J Biomed Sci. 2026 Jan 12;33:11. doi: 10.1186/s12929-025-01213-y (PMC12794471; doi:10.1186/s12929-025-01213-y)
Supplement: Supplementary file 1 — Supplementary Material 1 [file 12929_2025_1213_MOESM1_ESM.docx]

**Supplementary Material**

## **Comparison Mod-Cls-SE(2) with the rotational models**

## **Mod-Cls-SE(2) Architecture**


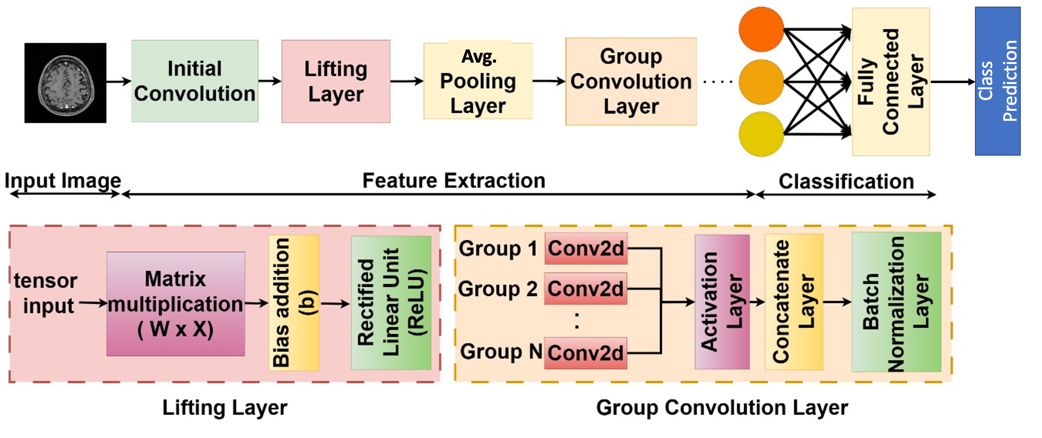


Fig 1. Mod-Cls-SE(2) architecture

The Mod-Cls-SE(2) architecture has been developed to address the challenges posed by geometric transformations, such as rotation and translation, particularly in image classification tasks. The core architecture is adapted from the original SE(2) group convolutional network proposed by [1]. Figure 1 illustrates the architecture of our Mod-Cls-SE(2). Each component layer is explained below.

- *Input Layer*

The model begins with an input layer that accepts a two-dimensional image tensor of shape $H\times W\times C$, where $H$and $W$ denote the spatial dimensions and $C$ is the number of image channels. This input is first processed through a cross-correlation between the input image $f$ and a convolution kernel $k$ in Euclidean space $\mathbb{R}^{2}$, mathematically defined as:

| $\left( k\star f \right)\left( x \right):=\int_{\mathbb{R}^{2}} k\left( x'-x \right)f\left( x' \right) dx'$ | (1) |
| --- | --- |

This operation extracts essential low-level spatial features such as edges and textures.

- *Lifting Layer*

In order to extend the feature representation into a space that incorporates rotation, the network employs a lifting layer. The function of this layer is to transform the input from Euclidean space to the roto-translation group space SE(2). This transformation is expressed through the group lifting correlation:

| $\left( k\tilde{\star}f \right)\left( g \right):=\sum_{c=1}^{N_{c}} \int_{\mathbb{R}^{2}} k_{c}\left( R_{\theta}^{-1}y-x \right)f_{c}\left( y \right) dy$ | (2) |
| --- | --- |

where $g=\left( x,\theta\right)$ denotes an element in SE(2), $R_{\theta}$ is the rotation matrix, $k_{c}$is kernel, and $N_{c}$ is the number of channels. This allows the network to encode both spatial and directional information.

- *Group Convolution Layer*

After lifting, the feature maps are processed by the group convolution layer, which generalizes standard convolution to operate over the SE(2) domain. The group convolution is formulated as:

| $\left( K\star F \right)\left( g \right):=\sum_{c=1}^{N_{c}} \int_{SE\left( 2 \right)} K_{c}\left( g^{-1}\cdot h \right)F_{c}\left( h \right) dh$ | (3) |
| --- | --- |

where $K_{c}$ is a kernel defined over SE(2), and $F_{c}$ is the lifted input. This operation ensures that the learned features remain equivariant to rotation and translation transformations.

- *SE(2) Multi-channel Feature Map*

At deeper layers, multiple SE(2) group convolution kernels are applied in parallel, producing a multi-channel feature map. The output of this layer is represented as:

| $F^{\left( l \right)}=\left( K_{1}^{\left( l \right)}\star F^{\left( l-1 \right)},K_{2}^{\left( l \right)}\star F^{\left( l-1 \right)},\ldots,K_{N_{l}}^{\left( l \right)}\star F^{\left( l-1 \right)} \right)$ | (4) |
| --- | --- |

where $K_{i}^{\left( l \right)}$ denotes the $i$-th kernel at layer $l$, and each output channel captures distinct rotation-aware features from the previous layer.

- *Average Pooling*

To transform the SE(2) representation back into a conventional 2D image, a projection layer is applied. This layer performs average pooling over the orientation axis, producing a rotation-invariant output:

| $f^{\left( l \right)}\left( x \right)=\underset{\theta\in[0,2\pi)}{\mathrm{avg}}F^{\left( l \right)}\left( x,\theta\right)$ | (5) |
| --- | --- |

This operation extracts the most prominent activation across all orientations for each spatial location $x$, allowing the network to retain the strongest response regardless of object orientation.

- *Output Layer and Activation Function*

Subsequent to these transformations, the model applies standard deep learning components. After each convolutional or group convolution layer, a matrix multiplication and bias addition are followed by a ReLU activation function to introduce non-linearity. Batch normalization is used to normalize the output across mini-batches, improving training stability. An average pooling layer then reduces the spatial resolution of the feature maps. The resulting features are flattened and passed through fully connected (dense) layers, culminating in an output layer with softmax activation, which produces a probability distribution over the target classes. The integration of SE(2)-equivariant mechanisms with conventional CNN operations is a key innovation in the field. The Mod-Cls-SE(2) exhibits robust feature extraction that is stable under both rotation and translation. This property renders it highly effective for classification tasks involving geometric variability.

## **HarmonicNet Architecture**

HarmonicNet [3] is a convolutional neural network-harmonics circular integrate which is designed to enhance training stability and feature learning efficiency through architectural innovations such as identity mapping and parallel side feature maps. These components facilitate smoother gradient flow and provide richer contextual information, contributing to faster convergence and improved model robustness during training. The use of average pooling further reduces computational complexity while preserving critical spatial information. However, despite these advantages, HarmonicNet lacks explicit mechanisms to handle geometric transformations such as rotations and translations. This limitation may hinder its performance in domains like medical imaging, where object orientation and spatial invariance are critical for reliable interpretation.

### **HoverNet Architecture**

HoverNet [4] is specifically designed for pixel-wise classification in medical images, leveraging both residual and dense decoder units to enhance feature representation and training stability. The network begins with a large 7×7 convolutional kernel to capture low-level spatial features, followed by residual connections that facilitate deep learning by mitigating vanishing gradient issues. Dense decoder units further refine the extracted features by integrating multi-scale contextual information. Upsampling operations restore the spatial dimensions for pixel-level prediction, while 1×1 convolutions and a final softmax layer enable precise classification. A major strength of the multiple number of channels lies in its combined use of skip and dense connections, which preserve fine-grained spatial details and improve classification accuracy. However, the model lacks explicit mechanisms for handling geometric transformations such as rotation and translation, which may limit its robustness in scenarios involving spatial variability.

## **Parameter Count Comparison**

| **Model** | **Number of Parameters** |
| --- | --- |
| Mod-Cls-SE(2) | 12,284,518 |
| VGG16 | 138,300,000 |
| VGG19 | 143,000,000 |
| HarmonicNet | 33,347,000 |
| HoverNet | 30,000,000 |
| ResNet50 | 23,989,124 |
| ResNet101 | 43,059,588 |

This table presents the number of parameters of the various models used in the experiments. In general, the greater the number of parameters in a model, the longer it takes for training and inference. Models with huge parameters, such as VGG16 and VGG19, require more calculations during the training and prediction process, which can increase the computation time. In contrast, models with fewer parameters, such as Mod-Cls-SE(2), tend to have faster training and inference times. Therefore, model selection should consider the balance between accuracy and the time efficiency required.

# **Comparison Mod-Seg-SE(2) with traditional models**

# **Mod-Seg-SE(2) Architecture**

Fig 2. Mod-Seg-SE(2) architecture

The proposed Mod-Seg-SE(2) architecture is designed to enhance segmentation performance, particularly in conditions where objects appear at various orientations and positions. It integrates the SE(2) group structure into the network to achieve rotation and translation equivariance while preserving spatial details necessary for dense prediction tasks like semantic segmentation. The overall network structure is illustrated in Figure 2.

The architecture begins with an input layer that processes a 2D image tensor of shape *H×W×C*, where *H* and *W* are the image dimensions and *C* is the number of channels. As described in Equation (1), the first convolutional layer applies a 3x3 kernel to extract foundational spatial features such as edges and textures. This operation is defined by the standard convolution equation, which has been previously outlined in Equation (1). To enable the network to be robust to geometric transformations, the extracted features are lifted into the SE(2) space using the lifting layer. This process allows the network to encode both spatial and rotational properties of features through the group lifting correlation, as described in Equation (2). Each input channel $f_{c}$ using rotated versions of the kernel $k_{c}$, producing features defined over SE(2) elements $g=\left( x,\theta\right)$.

To reduce the computational load while preserving essential information, an average pooling layer is applied to downsample the lifted features. Following this, the model utilizes group convolution layers that operate over the SE(2) space to preserve transformation-equivariant representations. The first group convolution is defined as described in Equation (3). This operation enables consistent feature learning across rotated and translated inputs. In subsequent stages, multiple group convolution kernels are applied in parallel, yielding a diverse set of orientation-sensitive feature maps. The output of this multi-kernel operation is represented by Equation 4. Each kernel $K_{i}^{\left( l \right)}$ contributes to a separate output channel, increasing the representational power of the layer. Between group convolutions, ReLU activation functions are used to introduce non-linearity, followed by the concatenate layers that act as skip connections. These layers fuse feature maps from earlier and deeper layers, helping to preserve spatial detail and facilitating better reconstruction in the decoding phase. Batch normalization is applied after convolutional operations to normalize activations and stabilize the training process. In the decoding phase, up-sampling layers restore the spatial dimensions of the feature maps to their original size. This is followed by GDeconv (Group Deconvolution) layers, which serve to refine the resolution-enhanced maps by applying inverse operations of group convolutions using learned filters. Batch normalization is again applied after GDeconv layers for regularization. Finally, the output layer applies a softmax activation function at the pixel level, generating a probability distribution for each pixel's class label, resulting in a dense and accurate segmentation map. The integration of SE(2) structures via lifting and group convolution layers, combined with standard deep learning components like skip connections and upsampling, allows Mod-Seg-SE(2) to achieve high accuracy and robustness in segmentation tasks with significant geometric variation.

# **U-Net Architecture**

The U-Net architecture [7] is a widely adopted model for image segmentation tasks due to its symmetrical encoder-decoder design and effective use of skip connections, which allow it to retain fine-grained spatial information lost during downsampling. By combining low-level features from shallow layers with high-level semantic information from deeper layers, U-Net achieves accurate pixel-level predictions and demonstrates strong performance in biomedical imaging applications. The use of 3×3 convolutions, max pooling, and transposed convolutions enables efficient feature extraction and resolution restoration, while a final 1×1 convolution followed by softmax activation ensures precise class assignment at the pixel level. A notable strength of U-Net is its ability to balance detail preservation and computational efficiency, which contributes to stable training and accurate segmentation.

### **NN U-Net Architecture**

The NN U-Net [8] enhances the traditional U-Net by incorporating deep supervision and skip connections, improving gradient flow, training speed, and segmentation accuracy. It uses convolutional layers with Leaky ReLU, max pooling for downsampling, and deconvolution for upsampling. Deep supervision ensures effective learning in deeper layers, while skip connections preserve spatial detail. However, like U-Net, it lacks built-in mechanisms for handling geometric transformations such as rotation and translation, limiting its robustness in orientation-variant tasks.

## **Parameter Count Comparison**

| **Model** | **Number of Parameters** |
| --- | --- |
| Mod-Seg-SE(2) | 4,499,356 |
| U-Net | 31,112,545 |
| NN U-Net | 7,771,873 |

This table shows the number of parameters for the three models used in the segmentation task. Models with a larger number of parameters, such as U-Net and NN U-Net, tend to have more layers and features, which affects the complexity of the model. The Mod-Seg-SE(2) model, with a smaller number of parameters, offers a lighter structure, which may affect the speed of training and inference. The number of parameters is directly related to the capacity of the model to learn more complex features, but also affects the time taken during training and inference.

# **Dataset And Preprocessing**

In this study, we evaluate the Mod-SE(2) model on a combination of MRI and other medical image modality datasets. These datasets cover both classification and segmentation tasks, with each task targeting different aspects of medical image analysis. Below is the breakdown of the datasets.

## **Classification**

For the classification, the datasets, including MRI Brain Tumors were used. To do a classification task, we need the input images and their corresponding classes. The MRI Brain Tumors (Public) dataset created by Msoud [10], contains the MRI scans of three types of brain tumors (Gliomas, Meningiomas, Pituitary, and No tumor). Looking at the dataset from a broader biological standpoint, it highlights the growing potential of using automated methods in neuro-oncology. We need to build a model that has a diagnostic approach by providing faster, more consistent, and unbiased analysis of medical images.

The MRI Brain Tumors (Private) dataset is collected from the National Taiwan University Hospital in Taiwan. Figure 3 illustrates an example of the MRI scans and their annotated tumor labels from the private dataset, highlighting the anatomical variability observed in clinical imaging. It contains the MRI scans of patients with various types of brain tumors, potentially including AVM (Arteriovenous Malformation), Meningioma, Metastases, Pituitary tumors, and Schwannoma. This dataset offers valuable insight into the structure and behavior of tumor tissues as seen through advanced imaging. MRI is a key tool in both biological research and clinical diagnostics because it can capture detailed images of soft tissues without the need for invasive procedures. An access to high-resolution MRI data allows researchers to study tumors at a larger scale, observing important features like uneven shapes, areas of dead tissue, and inconsistent tissue textures. These characteristics help shed light on the tumor environment, such as low oxygen zones or new blood vessel growth, which are all hallmarks of cancer. Since the MRI scans come from a private source, access is restricted, and researchers must be careful to follow ethical and privacy standards. This collection of scans provides a strong foundation for advancing our understanding of tumor representation and what they look like in real clinical settings. The private dataset reveals substantial variability in tumor position, size, and shape, presenting a significant challenge for accurately classifying the tumors into their respective categories.

Fig 3. Brain tumor MRI dataset (Private)

## **Segmentation**

For the segmentation task, the datasets containing Blood Cell, Skin Lesion, BraTS2020, and Brain Tumor MRI (Private) were used. To do a segmentation task, we need a pair of input images and the ground truth of the object. The Blood Cell Count and Detection (BCCD) dataset is used for cell type segmentation [11]. For the Blood Cell dataset, the annotated white blood cell types include lymphocytes, monocytes, eosinophils, and neutrophils. Segmenting blood cells in this dataset presents several intrinsic challenges stemming from the biological nature of the images and imaging conditions. The Skin Lesion dataset is used for skin lesion segmentation [12]. For the Skin Lesion dataset, the lesion types include malignant melanoma, benign melanocytic nevus, and seborrheic keratosis. Segmentation of skin lesions poses equally complex challenges, largely due to the high variability in lesion appearance and imaging conditions. The irregular and often asymmetric shapes of lesions, combined with the lack of clearly defined edges, further challenge segmentation accuracy. These factors collectively demand advanced segmentation models with strong spatial attention and noise-resilience capabilities.

BraTS2020 [10] dataset is used for brain tumor segmentation and serves as a widely recognized benchmark in medical imaging. It includes multimodal MRI scans, T1, T1c, T2, and FLAIR, of patients, along with expert-annotated ground truth masks for tumor subregions (enhancing tumor, tumor core, and whole tumor). The complexity of this dataset arises from tumor heterogeneity, irregular boundaries, and intensity non-uniformities across modalities, making it ideal for evaluating the robustness of segmentation models. In this study, BraTS2020 is employed alongside a private MRI brain tumor dataset to assess segmentation performance across varied tumor characteristics and imaging conditions.

MRI Brain Tumors (Private) datasets, which were also used in the classification task, are employed here as well for tumor segmentation. In our MRI brain dataset, the tumors are characterized by inconsistent sizes, unpredictable locations, and non-uniform shapes, all of which hinder robust classification into distinct categories. Figure 4 presents representative MRI scans from the private dataset alongside their corresponding ground truth segmentation masks, illustrating the anatomical variability of tumor appearances in real clinical cases. For details on these datasets, including the number of images and access links, please refer to the classification section above.

Fig 4. Brain tumor MRI dataset (Private) and their corresponding ground truth

# **Fine-scale feature detection**

We conducted an experiment to empirically demonstrate the functional advantage of the Mod-SE(2) in detecting objects under varying size conditions. Specifically, we evaluated the model’s ability to preserve feature activations across reduced object sizes (20×20, 10×10, and 5×5 pixels) and under spatial transformations, including translation and rotation. A synthetic dataset was used to ensure a fully controlled environment, allowing precise manipulation of object size and orientation. Figure 5 shows the design of the synthetic phantom image used throughout the experiment. By systematically reducing the object size and altering spatial attributes, this experiment investigates how well Mod-SE(2) maintains spatial sensitivity and robustness in feature detection, especially in early convolutional layers. This experimental validation using synthetic datasets supports the theoretical claims of the Mod-SE(2), demonstrating its improved robustness to changes in orientation and position. Unlike standard CNNs, which are not inherently invariant and equivariant to translation or rotation, Mod-SE(2) leverages group convolutions to maintain sensitivity to these transformations. The Mod-SE(2) exhibits robust sensitivity to object transformations, maintaining high feature activation even when objects undergo rotation or moderate downscaling, a capability where traditional CNNs fail. Mod-SE(2) was compared with VGG19 (traditional model) and Harmonics Net, which integrate circular harmonics. The comparison aims to evaluate how each model handles transformations and to see whether Mod-SE(2) offers advantages in detecting features.


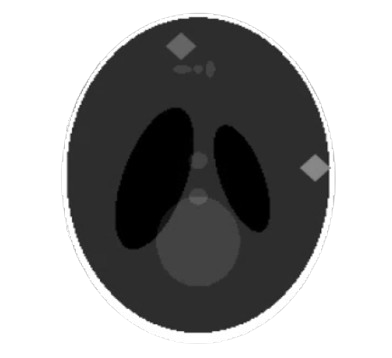


Fig 5. An example of synthetic image

We first tested the model using square objects sized 20×20 pixels. The dataset was constructed to prevent duplicate images across training, validation, and testing, and object locations were randomly varied to simulate translation and rotation. Figure 6 presents an example image from the 20×20-pixel dataset, highlighting object appearance across training, validation, and testing dataset. Figure 6a shows the training dataset, where the target object is embedded within a consistent anatomical background and subjected to various spatial transformations, including translations and rotations, to simulate diverse conditions. Figure 6b presents the validation set, which includes novel object positions not seen during training, and is used to evaluate the model’s ability to generalize spatially. Finally, Figure 6c displays the testing set, where the object appears in entirely unseen configurations, distinct from both the training and validation sets. After feeding the dataset into each model, we extracted feature maps from the first convolutional layer to analyze spatial sensitivity. Figure 7 illustrates the resulting feature activations. Mod-SE(2) produced strong activations (bright yellow regions), indicating its high sensitivity to object presence. In comparison, VGG and HarmonicNet were able to detect the object’s location but with noticeably lower activation intensity.

Fig 6. An example of a 20x20 dataset illustrating object appearance across different dataset partitions.
**(a)** Training dataset: the target object is embedded within a consistent anatomical background and subjected to spatial transformations such as translations and rotations to simulate diverse imaging conditions. **(b)** Validation dataset: contains novel spatial configurations not present in training, used to evaluate the model’s generalization to unseen object positions. **(c)** Testing dataset: features entirely new object configurations.


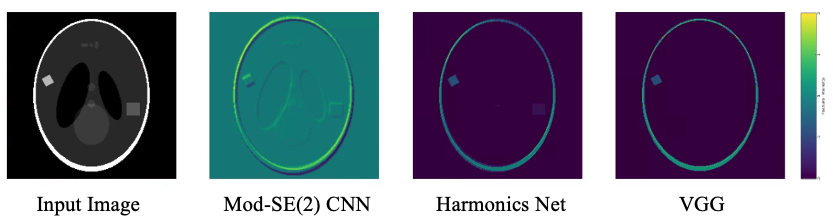


Fig 7. Feature map of the 20x20 dataset

Next, we reduced the object size to 10×10 pixels while maintaining the same experimental conditions. Figure 8 presents a sample image from the 10×10-pixel dataset, illustrating how object size affects spatial representation across the training, validation, and testing sets. Figure 8a shows the training set, where the target object is embedded at various positions within a reduced-scale object. Figure 8b includes validation images with novel object placements that are not present in training, allowing the evaluation of spatial generalization for smaller-scale features. Figure 8c depicts the testing set with entirely unseen object configurations. Due to the smaller object size, the visual prominence is diminished, challenging the ability of the model to localize and respond effectively. To assess the impact of this scale reduction, feature maps from the first convolutional layer were extracted and analyzed, revealing decreased activation levels in response to the smaller object, thereby highlighting the model’s reduced sensitivity to fine-scale features. When the object is reduced to 10x10 pixels, we can see that the activation on the feature map starts to decrease. As expected, the activation maps showed a decline in response intensity. Figure 9 displays the feature maps generated by each model. Mod-SE(2) retained moderate activation (green to blue-green tones), showing it could still localize the object with some loss of detail. HarmonicNet exhibited reduced activation, and VGG failed to detect the object altogether.

Fig 8. An example of a 10x10 dataset demonstrating the impact of reduced object size on spatial representation across dataset partitions. **(a)** Training dataset: the target object appears at various positions with reduced scale, embedded within a consistent background to simulate diverse spatial scenarios. **(b)** Validation dataset: includes novel object placements not seen during training, used to evaluate the model’s spatial generalization to smaller-scale features. **(c)** Testing dataset: features entirely new configurations, designed to assess the model’s robustness to fine-scale spatial variability.


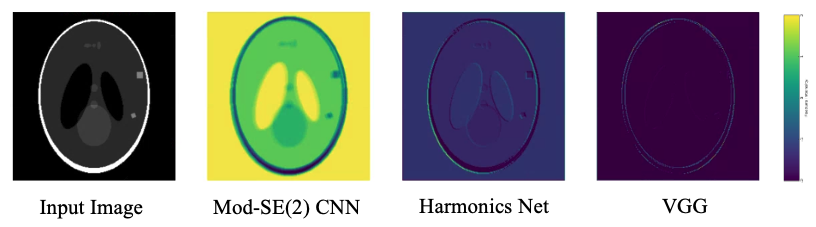


Fig 9. Feature map of the 10x10 dataset

To further examine the detection limits of each model, we decreased the object size to 5×5 pixels. Figure 10 presents an example from the 5×5-pixel dataset, illustrating the most extreme case of object size reduction across the training, validation, and testing sets. Figure 10a displays the training images, where the significantly smaller object is placed. Figure 10b shows the validation images with object placements not encountered during training, while Figure 10c illustrates testing samples with entirely new spatial configurations. At this minimal scale, the object becomes visually subtle and easily overshadowed by surrounding anatomical structures. At this size, Mod-SE(2) showed a marked drop in activation strength, indicating difficulty in detecting the object. Figure 11 shows the resulting feature maps, where both VGG and HarmonicNet completely failed to detect the phantom object, while Mod-SE(2) still maintained low but visible responses.

Fig 10. An example of a 5x5 dataset, representing the smallest object size reduction across dataset partitions. **(a)** Training dataset: includes images with the target object significantly reduced in scale, embedded within a consistent background. **(b)** Validation dataset: contains novel object placements not seen during training, testing the model’s ability to generalize spatially at minimal scale. **(c)** Testing dataset: features entirely unseen object configurations, further challenging model robustness.


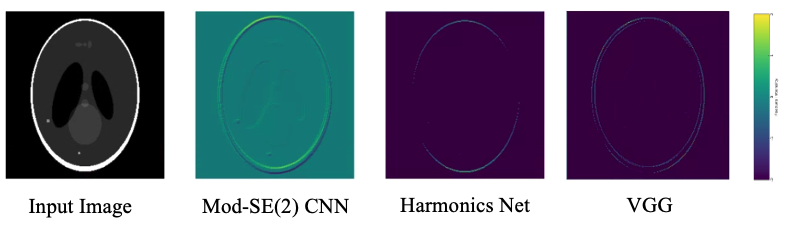


Fig 11. Feature map of the 5x5 dataset

Based on our experiments, we identified the minimum object size detectable by the proposed Mod-SE(2) model to be 5×5 pixels. The synthetic dataset was generated using images of 256×256 pixel resolution, corresponding to a field of view (FOV) of 128 mm, resulting in a pixel spacing of 0.5 mm/pixel. Accordingly, phantom objects with dimensions of 20×20, 10×10, and 5×5 pixels represent estimated physical sizes of 10 mm, 5 mm, and 2.5 mm, respectively. These results indicate that the Mod-SE(2) can robustly detect objects as small as 2.5 mm, confirm the Mod-SE(2) model’s strong spatial sensitivity and its capacity for fine-scale object recognition in medical imaging contexts.

# **Group Discretization in SE(2) Convolution**

Group discretization determines how many sampled orientations are used in the SE(2) group convolution. This hyperparameter controls the model’s rotational granularity, where a larger number of orientations increases geometric expressiveness but also increases computational cost. To better understand this trade-off, we evaluated Mod-Seg-SE(2) under three standard discretization choices: 4, 8, and 16 orientations. All experiments were conducted using the BraTS dataset for segmentation. The architecture, training schedule, and data preprocessing were kept identical and only the number of sampled orientations in the SE(2) group convolution kernels was changed. We report inference time (seconds per image) and Dice coefficient as performance metrics.

| **Group orientation** | **Inference time (seconds/image)** | **Segmentation Dice Score** |
| --- | --- | --- |
| 4 orientation | 0.0925 | 0.9272 |
| 8 orientation | 0.1099 | 0.9503 |
| 16 orientation | 0.1977 | 0.9531 |

These findings highlight a clear computational and accuracy trade-off, where 4 orientations offer the fastest inference but noticeably reduced segmentation quality due to limited rotational sensitivity. 8 orientations deliver the optimal balance, significantly improving accuracy over 4 orientations while maintaining practical inference speed suitable for clinical deployment.16 orientations provide the highest Dice score, but with nearly double the inference time of 8 orientations. This experiment supports the selection of 8 orientations as the most efficient and clinically appropriate group discretization level for Mod-SE(2), presenting the best balance between the model’s performance and inference time.

# **References**

[1] E.J. Bekkers, M.W. Lafarge, M. Veta, K.A.J. Eppenhof, J.P.W. Pluim, and R. Duits, “Roto-translation covariant convolutional networks for medical image analysis,” in Medical Image Computing and Computer Assisted Intervention – MICCAI 2018*,* Springer, Cham, pp. 440–448, 2018, https://doi.org/10.48550/arXiv.1804.03393

[2] L. Ali, F. Alnajjar, H. Jassmi, M. Gochoo, W. Khan, and M. Serhani, “Performance Evaluation of Deep CNN-Based Crack Detection and Localization Techniques for Concrete Structures,” Sensors, vol. 21, p. 1688, Apr. 2021, doi: 10.3390/s21051688.

[3] V. Sudha and D. Ganeshbabu, “A Convolutional Neural Network Classifier VGG-19 Architecture for Lesion Detection and Grading in Diabetic Retinopathy Based on Deep Learning,” Computers, Materials & Continua, vol. 66, pp. 827–842, Apr. 2020, doi: 10.32604/cmc.2020.012008.

[4] D. E. Worrall, S. J. Garbin, D. Turmukhambetov, and G. J. Brostow, “Harmonic Networks: Deep Translation and Rotation Equivariance,” 2017. [Online]. Available: https://arxiv.org/abs/1612.04642

[5] S. Graham et al., “Hover-Net: Simultaneous segmentation and classification of nuclei in multi-tissue histology images,” Med Image Anal, vol. 58, p. 101563, 2019, doi: https://doi.org/10.1016/j.media.2019.101563.

[6] Z. Duan, F. Wang, B. Wang, G. Luo, and Z. Jiang, “An Adapted ResNet-50 Architecture for Predicting Flow Fields of an Underwater Vehicle,” IEEE Access, vol. 12, pp. 66398–66407, 2024, doi: 10.1109/ACCESS.2024.3399077.

[7] A. Ahmad et al., “Human Gait Recognition Using Deep Learning and Improved Ant Colony Optimization,” Computers, Materials and Continua, vol. 70, pp. 2261–2276, Apr. 2022, doi: 10.32604/cmc.2022.018270.

[8] O. Ronneberger, P. Fischer, and T. Brox, “U-Net: Convolutional Networks for Biomedical Image Segmentation,” 2015. [Online]. Available: https://arxiv.org/abs/1505.04597

[9] N. McConnell, N. Ndipenoch, Y. Cao, A. Miron, and Y. Li, “Exploring advanced architectural variations of nnUNet,” Neurocomputing, vol. 560, p. 126837, 2023, doi: <https://doi.org/10.1016/j.neucom.2023.126837>.

[10] Aswaf: Brain Tumor Segmentation (BraTS2020) dataset (2020). https://www.kaggle.com/datasets/awsaf49/brats2020-training-data

[11] Msoud N.: Brain Tumor MRI Dataset (2020). <https://doi.org/10.34740/kaggle/dsv/2645886>

[12] Blahiri J.: BCCD dataset with mask: A blood cell detection dataset (2023). <https://www.kaggle.com/datasets/jeetblahiri/bccd-dataset-with-mask>

[13] Pivoshenko V.: Skin cancer lesions segmentation dataset (2023). <https://www.kaggle.com/datasets/volodymyrpivoshenko/skin-cancer-lesions-segmentation>
