# Supplementary figures and images for: Mod-SE(2): a geometric deep learning framework for brain tumor classification and segmentation in MRI images
Source: J Biomed Sci. 2026 Jan 12;33:11. doi: 10.1186/s12929-025-01213-y (PMC12794471; doi:10.1186/s12929-025-01213-y)

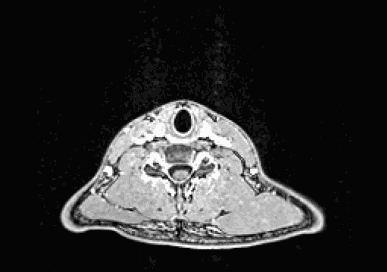

Supplement: Supplementary file 2 — Supplementary Material 2 [file 12929_2025_1213_MOESM2_ESM.gif]

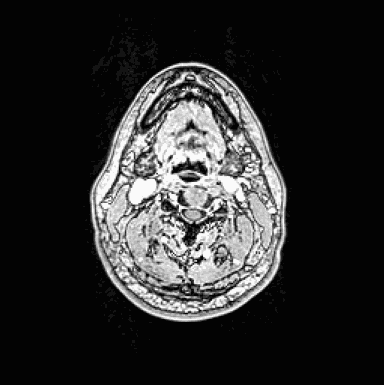

Supplement: Supplementary file 3 — Supplementary Material 3 [file 12929_2025_1213_MOESM3_ESM.gif]

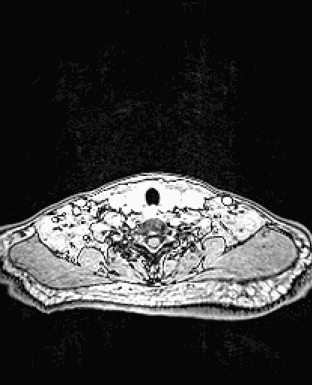

Supplement: Supplementary file 4 — Supplementary Material 4 [file 12929_2025_1213_MOESM4_ESM.gif]
