# Supplementary material for: Mod-SE(2): a geometric deep learning framework for brain tumor classification and segmentation in MRI images
Source: J Biomed Sci. 2026 Jan 12;33:11. doi: 10.1186/s12929-025-01213-y (PMC12794471; doi:10.1186/s12929-025-01213-y)
Supplement: Supplementary file 5 — Supplementary Material 5 [file 12929_2025_1213_MOESM5_ESM.docx]

***Mod-SE(2): A Geometric Deep Learning Framework for Brain Tumor Classification and Segmentation in MRI Images***

***Supplementary Videos***

Clara Lavita Angelina^1,2,3^**^†^**, Fu-Ren Xiao^3,10^**^†^**, Sunil Vyas^3,7^**^†^**, Pan-Chyr Yang^4,5,7,9,10*^, Hsuan-Ting Chang^1,2,6*^, Yuan Luo^3,7,8,9,10*^

*^1^Department of Electrical Engineering, National Yunlin University of Science and Technology, Yunlin, 64002, Taiwan.*

*^2^Graduate School of Engineering Science and Technology, National Yunlin University of Science and Technology, Yunlin, 64002, Taiwan.*

*^3^Institute of Medical Device and Imaging, National Taiwan University, Taipei, 10051, Taiwan.*

*^4^Department of Internal Medicine, National Taiwan University Hospital, Taipei, 100225, Taiwan.*

*^5^National Taiwan University Hospital, National Taiwan University, Taipei, 10051, Taiwan.*

*^6^Graduate School of Intelligent Data Science, National Yunlin University of Science and Technology, Yunlin, 64002, Taiwan.*

*^7^YongLin Institute of Health, National Taiwan University, National Taiwan University, Taipei, 10087, Taiwan.*

*^8^Department of Biomedical Engineering, National Taiwan University, Taipei, 10617, Taiwan.*

*^9^Taiwan International Bio Research Center, National Taiwan University, Taipei, 106319, Taiwan.*

*^10^Program for Precision Health and Intelligent Medicine, National Taiwan University, Taipei, 106319, Taiwan.*

Correspondence and Requests for materials should be addressed to:

*Corresponding author:

Yuan Luo, Ph.D.

Institute of Medical Device and Imaging,

College of Medicine

No. 1, Section 1, Ren'ai Rd, Zhongzheng District,

National Taiwan University

Taipei, Taiwan 10051

E-mail : [yuanluo@ntu.edu.tw](mailto:yuanluo@ntu.edu.tw)

Tel: +886-23123456

ext 288453

**Supplementary Video 1 (Supplementary File 2) .**  **A series of axial MRI brain scans from their 3D data depicting a case of Arteriovenous Malformation (AVM).** The AVM region is highlighted in yellow, showcasing its subtle and compact structure across multiple slices. The video emphasizes the model’s ability to localize and delineate small, irregularly shaped lesions, demonstrating its sensitivity to fine-scale anatomical variations that are often difficult to detect using conventional imaging methods.

**Supplementary Video 2 (Supplementary File 3). A series of axial MRI brain scans from their 3D data illustrating a case of Meningioma**. The tumor region is highlighted in **yellow**, providing visual localization of the lesion across multiple slices. The video demonstrates the spatial extent and anatomical positioning of the meningioma, aiding in the qualitative assessment of tumor morphology and model prediction alignment.

**Supplementary Video 3 (Supplementary File 4). A series of axial MRI brain scans from their 3D data illustrating a case of Pituitary.** The video enables clear visualization of the tumor location relative to surrounding anatomical structures, which are highlighted in **yellow**. The video aids in illustrating the tumor's size, shape, and growth pattern within the regions, supporting visual evaluation and model-based segmentation performance.
